# Preponderance of generalized chain functions in reconstructed Boolean models of biological networks

**DOI:** 10.1038/s41598-024-57086-y

**Published:** 2024-03-20

**Authors:** Suchetana Mitra, Priyotosh Sil, Ajay Subbaroyan, Olivier C. Martin, Areejit Samal

**Affiliations:** 1 Indian Institute of Science Education and Research (IISER) Mohali, Manauli, Punjab 140306 India; 2 The Institute of Mathematical Sciences (IMSc), Chennai, 600113 India; 3 Homi Bhabha National Institute (HBNI), Mumbai, 400094 India; 4 Université Paris-Saclay, CNRS, INRAE, Univ Evry, Institute of Plant Sciences Paris-Saclay (IPS2), 91405 Orsay, France; 5 Université Paris-Cité, CNRS, INRAE, Institute of Plant Sciences Paris-Saclay (IPS2), 91405 Orsay, France

**Keywords:** Gene regulatory networks, Boolean networks, Update rules, Chain function, Nested canalyzing function, Relative enrichment, Computational biology and bioinformatics, Computational models, Gene regulatory networks, Applied mathematics

## Abstract

Boolean networks (BNs) have been extensively used to model gene regulatory networks (GRNs). The dynamics of BNs depend on the network architecture and *regulatory logic rules* (*Boolean functions* (BFs)) associated with nodes. Nested canalyzing functions (NCFs) have been shown to be enriched among the BFs in the large-scale studies of reconstructed Boolean models. The central question we address here is whether that enrichment is due to certain sub-types of NCFs. We build on one sub-type of NCFs, the *chain functions* (or *chain-0 functions*) proposed by Gat-Viks and Shamir. First, we propose two other sub-types of NCFs, namely, the class of *chain-1 functions* and *generalized chain functions*, the union of the chain-0 and chain-1 types. Next, we find that the fraction of NCFs that are chain-0 (also holds for chain-1) functions decreases exponentially with the number of inputs. We provide analytical treatment for this and other observations on BFs. Then, by analyzing three different datasets of reconstructed Boolean models we find that generalized chain functions are significantly enriched within the NCFs. Lastly we illustrate that upon imposing the constraints of generalized chain functions on three different GRNs we are able to obtain biologically viable Boolean models.

## Introduction

Cellular decision making processes are governed by intricate gene regulatory networks (GRNs)^1^. Extensive efforts have been dedicated in the pursuit of understanding their underlying structure and dynamics^2–7^. The discrete state framework of ‘logical modeling’ pioneered by Stuart Kauffman^8,9^ and René Thomas^10,11^, stands out as a simple and effective way to mimic the dynamic behaviour of GRN. In the Boolean formalism, the state of the nodes (genes or other biological entities) are simplified to two different states—‘off’ or ‘on’.

In order to replicate key steady-state dynamical behaviour in living systems such as fixed points, Kauffman explored Random Boolean networks (RBNs)^8^. RBNs are defined by the inclusion of interactions (directed edges) between nodes (genes) chosen at random, accompanied by the assignment of random logical update rules at these nodes. However, extensive studies of biological networks, aided by recent advances in collection of large-scale data, have revealed that the network architectures of GRNs are far from being random, both in terms of their topology and the logic rules^4,6,7,12,13^. Since the beginning of the present century, there has been a significant surge in employing the Boolean framework to reconstruct GRNs from experimental biological data^14–20^. This trend has been propelled by the advancement in sequencing technology and increased computational capabilities, allowing not only the reconstruction of networks but also in the reproduction of gene expression patterns.

Deeper investigations into the use of Boolean functions (BFs) has unveiled that specific classes of functions, such as unate functions (UFs)^21^, canalyzing functions (CFs)^2^ and nested canalyzing functions (NCFs)^3,22^ exhibit distinct properties that inherently render them more suitable to be regarded as biologically meaningful when compared to random BFs. Notably, some of us in our previous work have shown that two classes of BFs, namely NCFs and read-once functions (RoFs) exhibit unexpected prevalence^13^ within a compiled reference biological dataset of 2687 BFs derived from reconstructed Boolean models of biological systems despite their theoretical fraction within the space of all BFs being minuscule. We also showed that NCFs and RoFs have the ‘simplest logic’ with respect to the complexity measures ‘average sensitivity’^23^ and ‘Boolean complexity’^24^ respectively. In a more recent work, some of us have also shown that biologically meaningful logics have distinct effect on the structural features of the state transition graph when compared to random logics^25^ .

In this study, our focus centers on two specific sub-types of NCFs. One is known as the chain function class that was introduced by Gat-Viks and Shamir in 2003^26^. They argued for the ubiquity of these functions in biological networks. Furthermore, in the same year, Kauffman *et al.*^3^ provided a simplified definition of the chain function class based on the canalyzing input values. Their work revealed that out of the 139 rules (or BFs) compiled by Harris *et al.*^27^, 132 were NCFs and amongst those 107 were chain functions. In 2011, Akutsu *et al.*^28^ proposed an alternative definition of chain functions based on the Boolean expression, where the variable signs and the subsequent operators are strictly interdependent and governed by a control pattern that is a sequence of ‘0’ and ‘1’. In our current work, we present an equivalent definition of Akutsu *et al.*^28^, but without having to introduce their control pattern. We also introduce a novel sub-type of NCFs, comprised of the duals of the functions in the class of chain functions. To distinguish the class of chain functions from this new class, we refer to the former as chain-0 functions or $ChF_0$, and the latter one as chain-1 functions or $ChF_1$. We then term the union of these two classes as ‘generalized chain functions’ and denote it by $ChF_U$. We first derive a formula to count the number of functions of a *k*-input $ChF_0$ (or $ChF_1$). Then we investigate the fraction of $ChF_0$ and $ChF_1$ within NCFs. Next we assess the presence of functions from these two classes in more extensive and contemporary datasets derived from reconstructed biological Boolean networks (BNs). For this analysis, we utilize three reference biological datasets: (a) BBM benchmark dataset^29^ which is the most recent and largest repository of regulatory logic rules from which we extract 5990 BFs, (b) MCBF dataset^13^, comprising of 2687 BFs, compiled previously by some of us from 88 published biological models, and (c) Harris dataset^27^, comprising of 139 BFs. Furthermore, we demonstrate the practical utility of these special sub-types of NCFs in the context of model selection. In prior work^30^, we have shown that confining BFs to NCFs can yield a substantial reduction in the space of plausible models. However, in that work we showed that even for networks with 18 nodes, the search space remained sufficiently vast (even when restricting to NCFs), making exhaustive search infeasible. Therefore we ask whether the generalized chain functions can further curtail the space of potential candidate models. To do so, we perform a comprehensive case study using three biological models^31–33^, and finally use relative stability as a constraint to select for models within ensembles that employ chain functions.

## Methods

### Boolean models of gene regulatory networks

A Boolean model of a GRN consists of nodes and directed edges where nodes correspond to genes and directed edges correspond to the regulatory interactions between them^2,8,9^. Genes in a BN are either in an upregulated (‘on’) or downregulated (‘off’) state. In a BN with *N* nodes, we denote the state of the *i*th gene at time *t* by $x_i(t)$, where $i \in \{1,2,\ldots ,N\}$ and $x_i(t) \in \{0,1\}$. The state of the network at time *t* can be given by a vector $\textbf{X}(t) = (x_1(t), x_2(t), \ldots , x_N(t))$. The temporal dynamics of a BN are dictated by the BFs (or *logical update rules* or *regulatory logic*) along with an update scheme (*synchronous*^2^ or *asynchronous*^11^ are among the most common and popular update schemes). In the synchronous update scheme all nodes of the BN are updated simultaneously. Mathematically, this may be expressed as $x_i(t+1) = f_i(x_i^{(1)}(t), x_i^{(2)}(t),\ldots , x_i^{(k)}(t))$ $\forall i \in \{1,2, \ldots , N\}$, where $f_i$ is the BF that acts on the *k* inputs to node *i*, $j \in \{1,2,\ldots ,k\}$ and $x_i^{(j)}(t) \in \{x_1(t), x_2(t), \ldots , x_i(t)\}$. This type of local dynamics takes the network from the state $\textbf{X}(t)$ to the state $\textbf{X}(t+1)$. Such a scheme leads to two kinds of emergent dynamics. In the first, the system reaches a state which on the next (and subsequent) update is left unchanged, corresponding to a *fixed point attractor*. In the second, the system cycles infinitely through a fixed set of states on successive updates, corresponding to a *cyclic attractor*. The states that converge to an attractor (including the attractor itself) comprise its basin of attraction. In multi-cellular organisms, one considers that the fixed point attractors provide the gene expression patterns characteristic of different cellular types^2^.

### Representations and properties of Boolean functions

#### Truth table and output vector

A BF *f* with *k* inputs (which we also refer to as a *k*-input BF) can be specified via a truth table with $k+1$ columns and $2^k$ rows, where the first *k* columns correspond to the states of the input variables and the $(k+1)$th column corresponds to the output values. Each row of the truth table is a unique combination of the states of *k* input variables with the $(k+1)$th entry corresponding to the output value associated with that input combination. The output column of the BF can also be considered as a binary vector with $2^{k}$ elements. The bias *P* of a BF is the number of 1*s* in its binary output vector. More explicitly, the bias of a BF with *k* inputs is the number of its $2^{k}$ possible input configurations that lead to an output equal to 1. Biologically, it is the number of possible ways in which *k* transcription factors come together so as to activate a target gene. It is sometimes convenient to specify that output vector via the integer whose binary decomposition is given by the entries of that vector. Note that for a *k*-input BF there are $2^{2^{k}}$ distinct BFs possible since the vector has $2^k$ entries.

#### Boolean function expression

A *k*-input BF can also be represented as a Boolean expression that combines Boolean variables (${x_i} \in \{0,1\}$) with the logical operators conjunction (or AND or $\wedge$), disjunction (or OR or $\vee$) and negation (or NOT or $\overline{x}_i$). For illustration $x_1 \wedge (\overline{x}_2 \vee x_3$) and ($x_3 \wedge x_1 \wedge x_2) \vee (x_3 \wedge \overline{x}_1 \wedge \overline{x}_2)$ are cases of 3-input BFs.

#### Operations on Boolean functions

- **Negation of variables**: Given a *k*-input BF *f*, one can consider the modified function where some of the input variables are negated. There are $2^{k}$ possible negation operations if we include the negation of none of the inputs. Such a negation may or may not lead to a new BF but preserves the bias of the BF. For example if $f_1 = x_1 \wedge (\overline{x}_2 \vee x_3)$ and $f_2 = \overline{x}_1 \wedge (\overline{x}_2 \vee x_3)$, then one function can be obtained from the other by negating the variable $x_1$.
- **Permutation**: Given a *k*-input BF *f*, a permutation operation is performed by permuting the variables in the BF’s logical expression. There are *k*! possible permutation operations when one includes the identity permutation. Such an operation may or may not lead to a new BF but it preserves the bias of the BF. For example if $f_1 = x_1 \wedge (\overline{x}_2 \vee x_3)$ and $f_2 = x_2 \wedge (\overline{x}_1 \vee x_3)$, then one function can be obtained from the other by permuting the variables $x_1$ and $x_2$.
- **Complementation**: Given a *k*-input BF *f*, a complementation operation replaces the 0*s* and 1*s* of the output column of the truth table with 1*s* and 0*s* respectively. In terms of the Boolean expression, this is equivalent to negating all the variables and changing the $\wedge$ and $\vee$ operators to $\vee$ and $\wedge$ operators respectively. For example, the BFs $f_1 = x_1 \wedge (\overline{x}_2 \vee x_3)$ and $f_2 = \overline{x}_1 \vee (x_2 \wedge \overline{x}_3)$ are complements of each other.

The notions of symmetry in Boolean functions and the properties therein have been previously explored in several works and have also been shown to have consequences for the network dynamics^34–36^.

### Theory

#### Nested canalyzing functions

##### Definition 1

(NCF) A *k*-input BF is *nested canalyzing*^3,37^ with respect to a permutation $\sigma$ on its inputs $\{1,2,\ldots ,k\}$ if:

$$\begin{aligned} f(\textbf{x}) = {\left\{ \begin{array}{ll} b_{1} \quad \text{if}\ x_{\sigma (1)} = a_{1},\\ b_{2} \quad \text{if}\ x_{\sigma (1)} \ne a_{1},x_{\sigma (2)} = a_{2},\\ b_{3} \quad \text{if}\ x_{\sigma (1)} \ne a_{1},x_{\sigma (2)} \ne a_{2},x_{\sigma (3)}= a_{3},\\ \vdots \\ b_{k} \quad \text{if}\ x_{\sigma (1)} \ne a_{1},x_{\sigma (2)} \ne a_{2},\ldots ,x_{\sigma (k)} = a_{k},\\ \overline{b}_{k} \quad \text{if}\ x_{\sigma (1)} \ne a_{1}, x_{\sigma (2)} \ne a_{2},\ldots , x_{\sigma (k)} = \overline{a}_{k}.\\ \end{array}\right. } \end{aligned}$$

where $\textbf{x} = (x_1, x_2, \ldots , x_k)$. In the above equation, $a_{1},a_{2},\ldots ,a_{k}$ are the canalyzing input values and $b_{1},b_{2},\ldots ,b_{k}$ are the canalyzed output values for inputs $x_{\sigma (1)},\ldots ,x_{\sigma (k)}$ in the permutation $\sigma$ of the *k* inputs. In other words the inputs are $x_{\sigma (1)},\ldots ,x_{\sigma (k)}$ where $x_{\sigma (1)}=a_1$ leads to the output $b_1$ and so on. The inputs $x_{\sigma (i)}$ can be said to be *canalyzing in* $a_i$ which could be either 0 or 1. Here, $\overline{a}_{k}$ and $\overline{b}_{k}$ are the complements of the Boolean values $a_k$ and $b_k$, respectively.

Alternatively, NCFs can be represented succinctly via the following Boolean expression^38^:

1 $$\begin{aligned} f(\textbf{x}) = X_{\sigma (1)}\odot (X_{\sigma (2)}\odot (X_{\sigma (3)}\odot \ldots (X_{\sigma (k-1)} \odot X_{\sigma (k)}) \ldots )) \end{aligned}$$

where $$X_{\sigma (i)} \in \{x_{\sigma (i)}, \overline{x}_{\sigma (i)}\}$$ and $$\odot \in \{\wedge , \vee \}$$.

The Boolean expression in Eq. (1) may also be written to regroup the successive OR and AND logical operators:

2 $$\begin{aligned} f(\textbf{x})= X_{\sigma (1)} \vee \ldots \vee X_{\sigma (k_1)} \vee ( X_{\sigma (k_1+1)} \wedge \ldots \wedge X_{\sigma (k_2)}\wedge (X_{\sigma (k_2+1)} \vee \ldots \vee X_{\sigma (k_3)} \vee (\ldots ))) \end{aligned}$$

or, if the first logical operator is an AND:

3 $$\begin{aligned} f(\textbf{x})= X_{\sigma (1)} \wedge \ldots \wedge X_{\sigma (k_1)} \wedge ( X_{\sigma (k_1+1)} \vee \ldots \vee X_{\sigma (k_2)} \vee (X_{\sigma (k_2+1)} \wedge \ldots \wedge X_{\sigma (k_3)} \wedge (\ldots ))) \end{aligned}$$

##### Definition 2

(Layer) Given a *k*-input NCF *f* with respect to a permutation $\sigma$ on its inputs $\{1,2,\ldots ,k\}$, we can make explicit the consecutive canalyzing output values as follows:

4 $$\begin{aligned} f(\textbf{x}) = {\left\{ \begin{array}{ll} b_{1} \quad \text{if}\ x_{\sigma (1)} = a_{1},\\ b_{1} \quad \text{if}\ x_{\sigma (1)} \ne a_{1},x_{\sigma (2)} = a_{2},\\ \vdots \\ b_{1} \quad \text{if}\ x_{\sigma (1)} \ne a_{1},x_{\sigma (2)} \ne a_2,\ldots ,x_{\sigma (k_1)}=a_{k_1}\\ b_{2} \quad \text{if}\ x_{\sigma (1)} \ne a_{1},x_{\sigma (2)} \ne a_2,\ldots ,x_{\sigma (k_1)} \ne a_{k_1}, x_{\sigma (k_1+1)}=a_{k_1+1} \\ \vdots \\ b_{2} \quad \text{if}\ x_{\sigma (1)} \ne a_{1},x_{\sigma (2)} \ne a_2,\ldots ,x_{\sigma (k_2)}= a_{k_2}\\ \vdots \\ b_{n} \quad \text{if}\ x_{\sigma (1)} \ne a_{1},x_{\sigma (2)} \ne a_2,\ldots ,x_{\sigma (k_{n-1}+1)}=a_{k_{n-1}+1}\\ \vdots \\ b_{n} \quad \text{if}\ x_{\sigma (1)} \ne a_{1},x_{\sigma (2)} \ne a_2,\ldots ,x_{\sigma (k_n)}=a_{k_n}\\ \overline{b}_n \quad \text{if}\ x_{\sigma (1)} \ne a_{1},x_{\sigma (2)} \ne a_2,\ldots ,x_{\sigma (k_n)}=\overline{a}_{k_n};~\text{where} ~ k_n=k \end{array}\right. } \end{aligned}$$

where, $b_{i+1} = \overline{b}_i$ for all $i \in \{1,2,\ldots ,n\}$ and $\textbf{x} = (x_1, x_2, \ldots , x_k)$. Note that in Definition 1 the subscripts of ${b}_i$ label the canalyzing input, and that in Definition 2 the subscripts label the layer number. The consecutive canalyzing inputs giving the same canalyzed output are grouped into what is referred to as a *layer*^39^. The number of inputs present in a layer can be termed the *layer-size* and the number of layers is called the *layer-number*. Hereafter, we will denote the layer-size of the *i*th layer as $m_i$, the layer-size of the last layer being $m_{last}$. In the notation of Eq. (4), $m_1=k_1, m_2=k_2-k_1, \ldots , m_{last}=k_n-k_{n-1}$.

(Note that $b_i$ in both Definitions 1 and 2 represents the canalyzed output but they have different subscripts in order to highlight the change in canalyzed output upon changing the layer in Definition 2).

An equivalent definition of the layer can be provided based on the logical expression of a NCF. Specifically, each successive set of inputs followed by the same type of operators (‘$\wedge$’ or ‘$\vee$’) constitutes a distinct layer (see Eqs. (2) and (3)). In other words, in the expression of a NCF, whenever the operator flips from AND ($\wedge$) to OR ($\vee$) or vice-versa, a new layer begins (including the preceding variable of the flipped operator). For example, given the NCF $x_1\wedge x_2\wedge (\overline{x}_3 \vee x_4)$, we immediately observe that it has two layers with $m_1=m_2=2$.

#### Relationship between bias, operators and layers

We now formalize the relationship between the bias and the sequence of operators in the Boolean expression (see Eq. (1)) for NCFs. In Nikolajewa *et al.*^38^, the authors encoded the sequence of operators in the Boolean expression of *k*-input NCF (see Eq. (1)) with $k-1$ bits, but did not relate their encoding to the bias *P* of the NCF. This relationship between the bias and operator sequence was implicitly first used in Subbaroyan *et al.*^13^ but was not stated explicitly. The bias *P* of a *k*-input NCF can be expressed via its binary representation with *k* bits where the least significant bit is 1 since NCFs have odd bias^13,38^. The first $(k-1)$ bits of this binary string encode the operator sequence that appears in the associated Boolean expression of NCFs (see Eq. (1)) such that the bits 0 and 1 encode the operators ‘$\wedge$’ and ‘$\vee$’ respectively. We explain the relationship in more detail in SI text, Property 1.1. Let us illustrate this relation via an example. Consider a 4-input NCF with bias $P=5$. The associated binary string representation of *P* is 0101. The Boolean expression for a 4-input NCF with $P=5$ is $X_{\sigma (1)} \wedge (X_{\sigma (2)} \vee (X_{\sigma (3)} \wedge X_{\sigma (4)}))$ where $X_{\sigma (i)} \in \{x_{\sigma (i)}, \overline{x}_{\sigma (i)}\}$. The operator sequence for this expression is $(\wedge , \vee , \wedge )$ (starting from the outermost operator to the innermost one), while the initial $(k-1)$ bits of the binary representation are 010. For any NCF, both the layer-number and the layer-size of each layer is uniquely determined by the pair $\{k,P\}$. The case of $m_{last}$ is particularly interesting and will be useful in explaining several of our results. Some of these properties are as follows:

1. For all $P\ne 1$, $m_{last}$ is independent of *k* (see SI text, Property 1.2), *i.e.*, it is not affected by the leading zeroes in the binary string of length *k* representing *P*.
2. The value of $m_{last}$ for any *k*-input NCF with bias $P = 4t + 3$ and bias $P' = 4t + 5$ are equal for any $t \in \mathbb{N}_0$ (see SI text, Property 1.3).
3. All bias *P* ($\ne 1$) values of a *k*-input NCF with $m_{last} = m$ can be expressed as $P = S\cdot 2^{m+1} + 2^{m} \pm 1$ for some $S \in \mathbb{N}_0$ (see SI text, Property 1.4)

#### Number of NCFs for a given number of inputs

Li *et al.*^39,40^ provided a formula for calculating the number of NCFs for any number of inputs *k*. Here, we present that formula in our notation. The number of *k*-input NCFs with bias *P* is given by:

5 $$\begin{aligned} |NCF|_{k,P} =\frac{2^k \cdot k!}{m_1!m_2!\ldots m_{last}!} \end{aligned}$$

The set $\{m_1,m_2,\ldots ,m_{last}\}$ is uniquely determined for any *k* and *P* as explained above. The total number of NCFs for a particular *k* can be obtained by summing Eq. (5) over all odd biases as follows:

6 $$\begin{aligned} |NCF|_k = \sum _{\begin{array}{c} 1 \le P \le 2^k - 1,\\ P \text{ odd} \end{array}} |NCF|_{k,P}= & {} \sum _{\begin{array}{c} 1 \le P \le 2^k - 1,\\ P \text{ odd} \end{array}} \frac{2^{k} \cdot k!}{m_1!m_2!\ldots m_{last}!} \\= & {} \sum _{\begin{array}{c} 1 \le P < 2^{k - 1},\\ P \text{ odd} \end{array}} \frac{2^{k+1} \cdot k!}{m_1!m_2!\ldots m_{last}!} \end{aligned}$$

Note that the layer-number and layer-length of each layer is invariant under complementation of the NCF as one can obtain the binary string representation of $2^k-P$ by replacing the 0 bits by 1 and vice versa in the binary string representation of *P*.

#### Chain functions

Chain functions were first introduced by Gat-Viks and Shamir^26^. They were found to be a sub-type of NCFs^3,28^. We begin with a definition given in Akutsu *et al.*^28^ and then move on to an alternative version which is of importance to us and then finally to the definition of Kauffman *et al.*^3^ (based on canalyzing inputs).

##### Definition 3

(Akutsu *et al.*^28^) A function *f* is a chain function with the control pattern $c_2=c_3=\ldots =c_{k_1}=0, c_{k_1+1}=1, c_{k_1+2}=\ldots =c_{k_2}=0, c_{k_2+1}=1,\ldots$ and input variable states of the form $x_1,x_2,\ldots ,x_k$ if and only if it has either of the following two forms:

$$\begin{aligned} f= & {} \overline{x}_1 \vee \ldots \vee \overline{x}_{k_1} \vee (x_{k_1+1} \wedge \ldots \wedge x_{k_2} \wedge (\overline{x}_{k_2+1} \vee \ldots \vee \overline{x}_{k_3} \vee (\ldots ))), \\ f= & {} x_1 \wedge \ldots \wedge x_{k_1} \wedge (\overline{x}_{k_1+1} \vee \ldots \vee \overline{x}_{k_2} \vee (x_{k_2+1} \wedge \ldots \wedge x_{k_3} \wedge (\ldots ))) \end{aligned}$$

where the former and the latter expressions correspond to $c_1 = 1$ and $c_1=0$ respectively.

This expression points out some unique properties of the chain functions:

1. Every time a control variable takes the value 1, a new layer (for definition of layer see Definition 2) begins.
2. At all but the last layer, a positive variable is followed by an AND ($\wedge$) operator, whereas a negative variable is followed by an OR ($\vee$) operator.

We now define chain functions using the binary representation of the bias instead of the control pattern in the following manner.

##### Definition

$\mathbf {3^*}$ Consider a bias *P* such that the first $k-1$ significant bits of the *k*-bit binary representation of *P* are $v_1=v_2=\ldots =v_{k_1}=0,v_{k_1+1}=v_{k_1+2}=\ldots =v_{k_2}=1,v_{k_2+1}=\ldots =v_{k_3}=0,\ldots$. A *k*-input BF with the ordered input variables $x_1, x_2,\ldots ,x_k$, is a chain function with that bias if and only if:

$$\begin{aligned} f=x_1 \wedge \ldots \wedge x_{k_1} \wedge \left( \overline{x}_{k_1+1} \vee \ldots \vee \overline{x}_{k_2} \vee \left( x_{k_2+1} \wedge \ldots \wedge x_{k_3} \wedge (\ldots )\right) \right) \end{aligned}$$

Similarly, when the first $k-1$ significant bits of the *k*-bit binary representation of *P* are $v_1=v_2=\ldots =v_{k_1}=1,v_{k_1+1}=v_{k_1+2}=\ldots =v_{k_2}=0,v_{k_2+1}=\ldots =v_{k_3}=1,\ldots$, a function will be a chain function with bias *P* if and only if:

$$\begin{aligned} f=\overline{x}_1 \vee \ldots \vee \overline{x}_{k_1} \vee \left( x_{k_1+1} \wedge \ldots \wedge x_{k_2} \wedge \left( \overline{x}_{k_2+1} \vee \ldots \vee \overline{x}_{k_3} \vee (\ldots )\right) \right) \end{aligned}$$

The relationship between the binary representation of the bias *P* and the layer structure of a chain function expression can be understood as follows. Consider a *k*-input chain function with bias *P* (*P* is odd since chain functions are a sub-type of NCFs, which have odd bias). Suppose, the binary representation of *P* is given by:

$$\begin{aligned} P_{Bin} = \underbrace{00\ldots 0}_{m_1} \underbrace{11\ldots 1}_{m_2}\underbrace{00\ldots 0}_{m_3}\ldots \underbrace{11\ldots 1}_{m_{last}-1}1 \end{aligned}$$

Then, the associated expression of a chain function will be of the following form

7 $$\begin{aligned} f = \underbrace{x_1 \wedge \ldots \wedge x_{k_1}\wedge }_{m_1} ( \underbrace{\overline{x}_{k_1+1} \vee \ldots \vee \overline{x}_{k_2} \vee }_{m_2} (\underbrace{x_{k_2+1} \wedge \ldots \wedge x_{k_3} \wedge }_{m_3}(\ldots (\underbrace{\overline{x}_{k_{n-1}+1} \vee \ldots \vee \overline{x}_k}_{m_{last}})))) \end{aligned}$$

where, $m_1=k_1$, $m_2 = k_2-k_1$, $m_3 = k_3-k_2$, ..., and $m_{last} = k-k_{n-1}$. Note that other similar expressions where exactly one variable in the last layer is negated are also chain functions for the same bias *P*. Let us illustrate this via a specific example of a 6 input chain function with bias 7 with respect to an ordering on its inputs $\{x_1, x_2,\ldots , x_6\}$. The 6 bit binary representation of the integer 7 is ‘000111’ and therefore $v_1 = v_2 = v_3 = 0$ and $v_4 = v_5 = 1$. Then, the expression of the associated chain function could be any of the following:

$$\begin{aligned} f_1= & {} x_1 \wedge x_2 \wedge x_3 \wedge (\overline{x}_4 \vee \overline{x}_5 \vee \overline{x}_6)\\ f_2= & {} x_1 \wedge x_2 \wedge x_3 \wedge (\overline{x}_4 \vee \overline{x}_5 \vee x_6)\\ f_3= & {} x_1 \wedge x_2 \wedge x_3 \wedge (\overline{x}_4 \vee x_5 \vee \overline{x}_6)\\ f_4= & {} x_1 \wedge x_2 \wedge x_3 \wedge (x_4 \vee \overline{x}_5 \vee \overline{x}_6)\\ \end{aligned}$$

In the last layer, there are $m_{last}$ variables connected by $m_{last} - 1$ operators. Therefore, the ‘last’ variable $x_6$ which is not followed by any operator can be either positive or negative. Thus, $f_1$ and $f_2$ are consistent with the definition of chain functions. Note that $f_3$ and $f_4$ in the above equations may appear inconsistent with the definition of chain functions in that the operators associated with the positive variables $x_5$ and $x_4$ respectively are both $\vee$ operators. However, since all the variables in this last layer are connected by the $\vee$ operator, one is allowed to exchange variables along with their sign within that layer without altering the resulting BF. In this manner, the positive variables may be moved to the last position, thereby restoring the apparent inconsistency between $f_3$ and $f_4$.

Lastly, Kauffman *et al.*^3^ defined the chain functions as a specific constraint on the canalyzing inputs of the NCFs as follows:

##### Definition 4

(Kauffman *et al.* 2003) A *k*-input chain function is a *k*-input NCF where the first $(k-1)$ canalyzing input values are 0. The last input is canalyzing in both 0 and 1.

Henceforth, we will refer to these chain functions as $ChF_0$ since their canalyzing input values are 0. That viewpoint opens up other options for constraining NCFs. In particular, it is natural to consider having the first ($k-1$) canalyzing input values be 1 instead of 0; we then obtain a new sub-type of NCFs as will now be presented.

#### Introduction of chain-1 functions

##### Definition 5

A *k*-input chain-1 function ($ChF_1$) is a *k*-input NCF where the first $k-1$ canalyzing input values are 1 and the last input is canalyzing in both 0 and 1.

Just as for $ChF_0$ functions, one can provide multiple equivalent definitions of chain-1 functions. Here we provide one analogous to that in Definition 3*.

##### Definition 6

A *k* input chain-1 function with bias *P*, where the first $k-1$ significant bits of the *k*-bit binary representation of *P* are $v_1=v_2=\ldots =v_{k_1}=0,v_{k_1+1}=v_{k_1+2}=\ldots =v_{k_2}=1,v_{k_2+1}=\ldots =v_{k_3}=0,\ldots$, with the ordered input variables $x_1, x_2,\ldots ,x_k$ is given by

$$\begin{aligned} f=\overline{x}_1 \wedge \ldots \wedge \overline{x}_{k_1} \wedge \left( x_{k_1+1} \vee \ldots \vee x_{k_2} \vee \left( \overline{x}_{k_2+1} \wedge \ldots \wedge \overline{x}_{k_3} \wedge (\ldots )\right) \right) \end{aligned}$$

Similarly, when the first $k-1$ significant bits of the *k*-bit binary representation of *P* are $v_1=v_2=\ldots =v_{k_1}=1,v_{k_1+1}=v_{k_1+2}=\ldots =v_{k_2}=0,v_{k_2+1}=\ldots =v_{k_3}=1,\ldots$, the chain function will be of the form

$$\begin{aligned} f=x_1 \vee \ldots \vee x_{k_1} \vee \left( \overline{x}_{k_1+1} \wedge \ldots \wedge \overline{x}_{k_2} \wedge \left( x_{k_2+1} \vee \ldots \vee x_{k_3} \vee (\ldots )\right) \right) \end{aligned}$$

**Figure 1:**
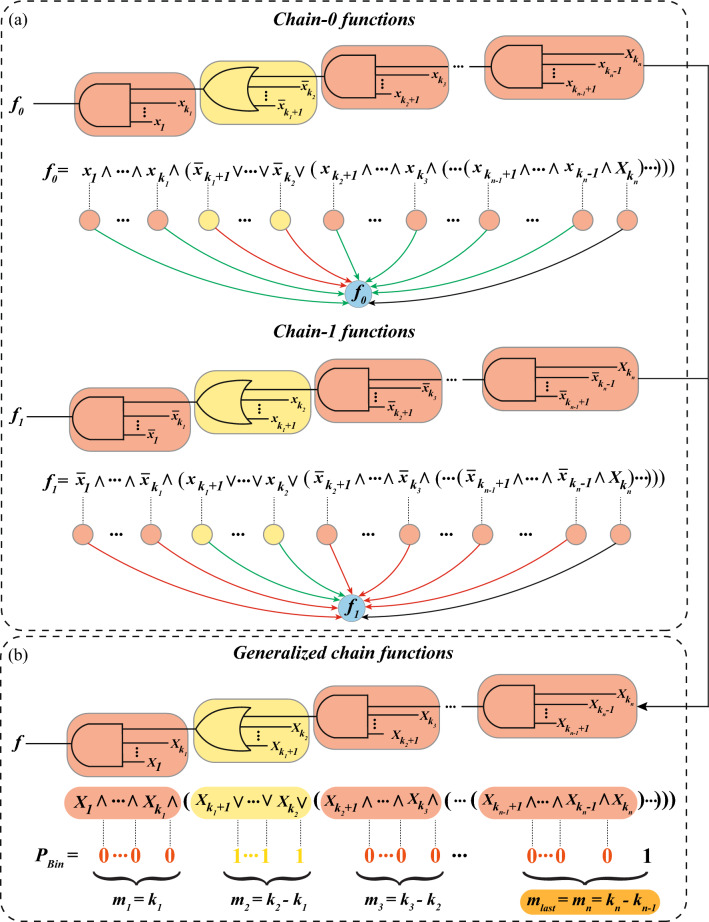
Relationship between layers, operators, variable signs and Boolean string representation of bias. The two circuit diagrams in **(a)** correspond to chain-0 ($f_0$) and chain-1 ($f_1$) functions respectively with bias $P<2^{k-1}$ and with a odd number of layers, where $k=k_n$. Rounded rectangular boxes with alternating colors correspond to successive layers. The schematics below the expressions of $f_0$ and $f_1$ denote the interactions driving the output of the BF. Here, the ‘green’ and ‘red’ arrows indicate ‘activatory’ (positive) and ‘inhibitory’ (negative) regulation respectively. The black arrows indicate regulation of either nature. Top part of **(b)** corresponds to the logic circuit diagram of the generalized chain function with bias $P<2^{k-1}$ and with an odd number of layers. Here, $X_i \in \{x_i, \overline{x}_i\}$ for all $i\in \{1,2,\ldots ,k_n\}$ with the constraint that the sign of all the variables in a given colored layer be the same (but altering from one layer to the next), with the exception of last layer. Note that in chain-0 (and chain-1) functions, only one input in the last layer ($i \in \{k_{n-1}+1, \ldots , k_n\}$) can be either $x_i$ or $\overline{x}_i$ (as is explained in the** Methods** section) and is hence denoted by $X_i$. The input $k_n$ was chosen to be capitalized arbitrarily. The Boolean expression form of the function is provided below the circuit diagram and layers are colored accordingly. The associations of operators and binary representation of the bias ($P_{Bin}$) are shown ($\wedge$ with 0 and $\vee$ with 1). $m_i$ correspond to the layer-size of the *i*th layer for all $i\in \{1,2,\ldots ,k_n\}$.

Clearly, the Boolean expressions of $ChF_1$ are similar to those of $ChF_0$ and one can obtain a $ChF_0$ from a $ChF_1$ and vice versa by either flipping the sign of all the variables (negating all the variables) or flipping all the operators (i.e., replacing $\wedge$ with $\vee$ and vice versa). By definition, both classes $ChF_0$ and $ChF_1$ are subsets of NCF and thus have odd bias. From $k=3$ onwards, they also form completely disjoint classes (see SI text, Property 2.1). We will refer to the union of $ChF_0$ and $ChF_1$ as **generalized chain functions** or $ChF_U$. Figure 1 is a schematic for the intuitive understanding of the chain-0, chain-1 and generalized chain functions.

### Reference biological datasets used for statistical analyses

In this section, we describe the 3 reference biological datasets containing the BFs that are used to quantify the abundance and enrichment of the $ChF_0$, $ChF_1$, $ChF_U$ and *non*-$ChF_U$ NCF types.

- **BBM benchmark dataset**: This dataset was adapted from the BBM benchmark dataset^29^, which consisted of 219 models of Boolean GRNs. Of these, we selected only the manually reconstructed ones, amounting to total 134 models. From those 134 models consisting of 6045 BFs, we extracted 5990 BFs (regulatory logic rules) when restricting to cases having at most 10 inputs.
- **MCBF dataset**: This dataset was published in Subbaroyan *et al.*^13^. This was downloaded from the Github repository https://github.com/asamallab/MCBF. It consists of 2687 BFs recovered from 88 manually reconstructed discrete models of Boolean GRNs. Here as well we restricted ourselves to BFs with at most 10 inputs per BF, leading to 2682 BFs.
- **Harris dataset**: This dataset is published in Harris *et al.*^3,27^. It consists of 139 BFs. This dataset has only BFs with 5 or less inputs and has been used exhaustively.

In both the MCBF and BBM benchmark datasets, the number of BFs for any number of inputs greater than 10 was too low to draw meaningful inferences from statistical significance tests and hence we did not include such cases in our study.

### Relative enrichment and associated $\varvec{p}$-values

The NCFs have been shown to be enriched in the space of all BFs and even within UFs and RoFs^13^. To test whether the enrichment of the NCF itself is due to its sub-types, namely, $ChF_0$, $ChF_1$ or $ChF_U$, we adapt relative enrichment and associated statistical significance test presented in Subbaroyan *et al.*^13^. Note that this significance is unrelated to the least significant bit spoken about earlier. Consider a type of BF *T* and one of its sub-types, say $T_s$. We are interested in relative enrichment of the sub-type $T_s$ within its englobing type *T* when considering a particular biological dataset. For instance we can examine the case where $T=$ NCF and $T_s = ChF_1$ (or any sub-type of NCF). We define the relative enrichment for a given number of inputs *k* by $E_R=(f_{s,1}/f_1)/(f_{s,0}/f_0)$ where $f_{s,1}$ and $f_1$ are the fraction of BFs belonging to $ChF_1$ and NCF respectively in the considered biological dataset, and $f_{s,0}$ and $f_0$ are the fractions of all *k*-input BFs belonging to $ChF_1$ and NCF respectively ($f_1$ and $f_0$ are both fractions of the NCF however, within the given dataset and within all possible *k*-input functions respectively). The type $T_s$ is relatively enriched within *T* only if $E_R > 1$, and not relatively enriched otherwise. However, the relative enrichment may be due to chance and must be shown to be statistically significant as we explain in the following section. In typical cases the biological dataset will have an over representation of BFs of type *T* and $T_s$ but by considering the relative enrichment we can test the hypothesis that this last enrichment is driven solely by the property of being in *T*; if the relative enrichment of $T_s$ within *T* is statistically inconsistent with the value 1, then we can reject the hypothesis. In particular, if $E_R$ is large, then there must be other factors than ‘belonging to *T*’ driving this relative enrichment.

#### Statistical significance and $\varvec{p}$-values

Our null hypothesis $H_0$ corresponds to assuming that although there is a selection for *T* the elements that are drawn within *T* have a uniform probability, that is members of $T_s$ are not more probable than the other elements of *T*. Consider then drawing a sample of BFs size *M* under $H_0$. If it leads to $M_T$ elements in *T* as in the reference biological dataset, the distribution of the number of elements in $T_s$ is known. Specifically, the probability to have *m* elements in $T_s$ is given by:

$$\begin{aligned} \left( \begin{array}{l}M_T \\ m\end{array}\right) (f_R)^m\left( 1-f_R\right) ^{M_T-m} \end{aligned}$$

where $f_R$ is the ratio of the sizes of $T_s$ and *T*. The desired *p*-value is then just the sum of all such probabilities under the condition that *m* is larger or equal to the number of $T_s$ elements in the reference biological dataset. The code for computing these *p*-values is available in our GitHub repository.

### Model selection using relative stability

Biologically meaningful types of BFs that occupy an extremely small fraction in the space of all BFs (such as $ChF_U$) can severely restrict the number of Boolean models for a given network structure—each node of the network may be constrained to belong to that type. We use the methodology developed in Zhou *et al.*^31^ and Subbaroyan *et al.*^30^ to generate a set of biologically plausible ensemble of models starting from a GRN with signed interactions and cell states (biological fixed points). That procedure involved applying several constraints on the truth table: (1) fixed point constraints; (2) biologically meaningful BFs; (3) BFs that obey the signs of the interactions in the network architecture. Constraint (1) ensures that all the models in the resulting ensemble recover the expected biological fixed points (fixed points that correspond to the cell states). However, this constraint does not guarantee the absence of spurious attractors. Constraint (2) restricts the type of BFs to biologically meaningful ones such as NCFs or RoFs^13^. In this work, we impose the $ChF_0$, $ChF_1$ and $ChF_U$ functions as our biologically meaningful types of BFs for different biological models. Constraint (3) forces the imposed biologically meaningful BFs to obey the signs of the interactions that have been observed experimentally. This procedure enables the generation of biologically plausible ensembles on which we can perform model selection using relative stability constraints as we explain later.

#### Relative stability and the mean first passage time

The relative stability of a pair of cell states (attractors) quantifies the propensity to transition from one cell state to the other versus in the opposite direction. Several measures of relative stability have been introduced in the literature^30,31,41^ of which the Mean First Passage Time (MFPT) captures best the directional aspect of state transitions. Here we succinctly present the mathematical framework proposed by Zhou *et al.*^31^ to define the relative stability of a pair of cell states. To begin, those authors extend the Boolean dynamics to render them stochastic. Mathematically, this change is specified by an $2^N$ $\times$ $2^N$ transition matrix (*N* being the number of nodes in the network):

8 $$\begin{aligned} \mathbf {T^{*}} = (1-\eta )^{N} \textbf{T} + \textbf{P} \end{aligned}$$

where $\textbf{T}$ and $\textbf{P}$ are the matrices representing the deterministic and stochastic components of the dynamics respectively. $T_{lm}$ are the entries of the deterministic matrix $\textbf{T}$, such that $T_{lm}=1$ if updating the state *m* via BFs $\textbf{F} = \{f_1, f_2, \ldots , f_N\}$ gives the state *l* (here *l*, *m* $\in \{0,1,\ldots ,2^{N} -1\}$ and 0 otherwise). $P_{lm}$ are the entries of the *perturbation* matrix $\textbf{P}$ such that $P_{lm}$ is the probability that a noise $\eta$ alone drives the transition from state *m* to state *l*. To be explicit, $P_{lm}$ is defined via:

$$\begin{aligned} P_{lm}= {\left\{ \begin{array}{ll} \eta ^{\text{d}(l,m)} (1- \eta )^{N - \text{d}(l,m)} &{} \text{if~} l \ne m \\ 0 &{} \text{if~} l = m\\ \end{array}\right. } \end{aligned}$$

where $\text{d}(l, m)$ is the Hamming distance between *l* and *m*. (Note that the Hamming distance between two distinct states of the network quantifies how different their gene expression patterns are; since values are Boolean, the distance between distinct states ranges from 1 to *N*, where 1 and *N* correspond to highly similar and different network states respectively). In brief, if the noise ($\eta$) does not alter the state of the network, then one applies the deterministic dynamics. The number of steps along a state space trajectory starting at state *m* and terminating at the first occurrence of *l* in a stochastic process is called the first passage time from state *m* to *l*. Its average over a large number of trajectories is then the MFPT from *m* to *l* and is denoted by $M_{lm}$. We use a stochastic method proposed in Subbaroyan *et al.*^30^ to compute the MFPT. If *u* and *v* denote 2 biological fixed points (cell states), then the MFPT $M_{uv}$ is the average of the number of time steps taken over a large number of trajectories starting at state *v* and evolved iteratively under the above-mentioned dynamics till state *u* is reached. Finally, Zhou *et al.*^31^ define the relative stability of cell state *u* compared to cell state *v* via:

9 $$\begin{aligned} RS_{MFPT}(u, v) = \frac{1}{M_{uv}} - \frac{1}{M_{vu}} \end{aligned}$$

$RS_{MFPT}(u, v) > 0$ if cell state *u* is more stable than cell state *v* and this condition is denoted by the inequality $u > v$. This inequality is also referred to as the ‘hierarchy’ associated with the pair of cell states. In this work, the noise intensity parameter value $\eta$ is set to 0.05; furthermore, 3000 state space trajectories of the first passage times are averaged over to obtain our estimates of each MFPT. Note that it has been previously shown^30^ that MFPTs and the hierarchies of pairs of cell states obtained using MFPT are relatively insensitive to small deviations of noise values from 0.01.

#### Biological models used to illustrate model selection

As case studies to illustrate model selection using relative stability (via MFPTs), we choose the 3 GRNs explained below.

- **Pancreas cell differentiation model:** The Pancreas cell differentiation network^31^ is a reconstructed GRN that controls the differentiation of cells in the pancreas. This model consists of 5 genes and 13 edges. This Boolean model has 3 fixed points corresponding to the cell types: Exocrine, $\beta /\delta$ cell progenitor and $\alpha$/PP cell progenitor (see SI Table S1).
- ***Arabidopsis thaliana root stem cell niche (RSCN-2010 model)***: The RSCN-2010 Boolean model (*model A* in Azpeitia *et al.*^32^) is a reconstructed Boolean GRN that controls the differentiation of cells in the root stem cell niche (RSCN) of *Arabidopsis thaliana*. The RSCN is located in the root tip of the plant. The 2010 BN has 9 nodes and 19 edges. The BF at each gene of this model is provided in SI Table S2. This Boolean model has 4 fixed points corresponding to the cell types: Quiescent center (QC), Vascular initials (VI), Cortex-Endodermis initials (CEI) and Columella epidermis initials (CEpI) (see SI Table S3). The BF for the AUX node for all our computations derived from this model is the same as the one provided in Velderrain *et al.*^42^.
- ***Arabidopsis thaliana root stem cell niche (RSCN-2020 model)***: The RSCN-2020 Boolean model^33^ is the latest reconstructed Boolean GRN that controls the differentiation of cells in the root stem cell niche (RSCN) of *Arabidopsis thaliana*. The 2020 BN has 18 nodes and 51 edges. The BF at each gene of this model is provided in SI Table S4. This model has 6 biological fixed points corresponding to the cell types: Quiescent center (QC), Cortex-endodermis initials (CEI), Peripheral Pro-vascular initials (P. Pro-vascular PD), Central Pro-vascular initials (C. Pro-vascular PD), Transition domain (C. Pro-vascular TD2) and Columella initials (Columella 1) (see SI Table S5).

#### Relative stability constraints for three biological GRNs

Using relative stability constraints derived from the published literature (as inequalities), we impose that Boolean models in the biologically plausible ensemble satisfy those constraints. Provided below are the known relative stability constraints for the 3 different biological models considered here. For the Pancreas cell differentiation model, the 3 relative stability constraints^31^ are: (1) Exocrine < $\alpha$/PP progenitor; (2) Exocrine $<\beta /\delta$ cell progenitor; and (3) $\alpha$/PP progenitor $<\beta /\delta$ cell progenitor. For the RSCN-2010 model, the 3 relative stability constraints^32,42^ are: (1) QC < VI; (2) QC < CEI; and (3) QC < CEpI. For the RSCN-2020 model, the 6 relative stability constraints^30,33^ are: (1) QC < CEI/EndodermisPD; (2) QC < P.ProvascularPD; (3) QC < C.ProvascularPD; (4) QC < C.ProvascularTD2; (5) QC < Columella1; and (6) C.ProvascularPD < C.ProvascularTD2. For each of the 3 biological models, we perform an exhaustive search over their biologically plausible ensembles for models that obey the above expected relative stability constraints. Additionally, we ‘select’ a model only if the sum over the sizes of the basin of attraction of the biological fixed points for that model is at least as large as that same sum for the original Boolean model to penalize models having spurious attractors.

## Results

### Properties of chain-0 and chain-1 functions

We now introduce some properties of the chain-0 ($ChF_0$) and chain-1 ($ChF_1$) functions based on the 3 operations on BFs described in **Methods**. These properties will be useful in counting the number of *k*-input $ChF_0$ (or $ChF_1$) with bias *P* as we shall see in the following section. We explain these properties here for $ChF_0$ only, but they hold true for $ChF_1$ as well.

**Figure 2:**
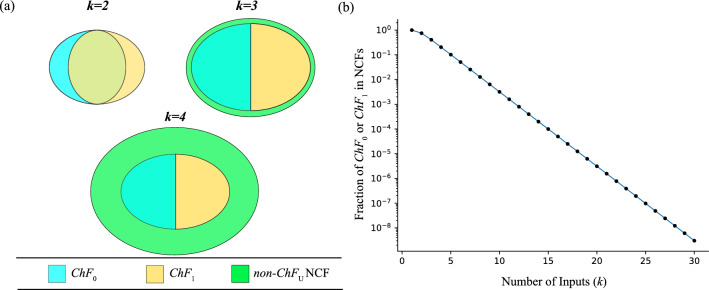
Fraction of chain-0 (or chain-1) functions within NCFs for different number of inputs. **(a)** Venn diagram of the space of $ChF_0$, $ChF_1$ within the NCFs. $k=1$ is not shown as $ChF_0$, $ChF_1$, $ChF_U$ and NCFs are identical. For $k=2$, the $ChF_0$ and $ChF_1$ do not completely overlap, and all NCFs are $ChF_U$s. For $k\ge 3$ the $ChF_0$ and $ChF_1$ sets become disjoint. **(b)** The fraction of $ChF_0$ (or $ChF_1$) within NCFs (*y*-axis) is plotted as a function of the number of inputs *k* (*x*-axis). The *y*-axis has a logarithmic scale. The linear trend (in the semi-log plot) suggests that the fraction of $ChF_0$ (or $ChF_1$) in NCF is an exponentially decreasing function of *k*.

1. A permutation of any chain-0 function is a chain-0 function. Permuting a $ChF_0$ shuffles the subscripts of the variables in its Boolean expression. Such an operation preserves the sequence of the operators ($\wedge$, $\vee$) and of the signs ($x_i$ versus $\overline{x}_i$) in that Boolean expression, thereby resulting in a $ChF_0$.
2. Negating variables in a chain-0 function may or may not produce a chain-0 function. A negation operation performed on any set of variables that are not in the last layer of a $ChF_0$ does not result in a $ChF_0$ since the signs of the negated variable and the operator following it become inconsistent with the definition of the $ChF_0$. For the last layer, if variables are linked by $\wedge$ (or $\vee$) operator, then at least $m_{last}-1$ variables must be positive (or negative). Thus only some negations in that layer may lead to a $ChF_0$ (see **Methods**).
3. The complement of a chain-0 function is a chain-0 function. The complementation operation simultaneously flips both the signs of all the variables (positive variables become negative and vice versa), and also the conjunction and disjunction operators ($\wedge$ operators are replaced by $\vee$ and vice versa). Such an operation preserves the sign-operator relations that characterize the $ChF_0$.
4. $ChF_0$ and $ChF_1$ form disjoint classes within the NCFs if and only if $k \ge 3$ (see SI text, Property 2.1 for proof, see Fig. 2a for illustration).
5. Permuting variables within the same layer does not alter the function (this is of course with exception of the last layer).

### Counting the number of chain-0, chain-1 and generalized chain functions

Gat-Viks and Shamir have provided a formula to count the number of $ChF_0$ for a given number of inputs based on a recursive approach^26^. We take a different approach and first count the number of *k*-input $ChF_0$ at bias *P*, using which we count the number of $ChF_0$ by summing over all odd biases. To count the number of *k*-input $ChF_0$ for a given bias *P*, we need to compute all permutations and negations of a reference chain-0 function that yield all the distinct $ChF_0$ with bias *P* (this suffices to obtain all $ChF_0$ with bias *P* as all NCFs with bias *P* form a single equivalence class under permutations and negations of variables^13^). The number of ways to permute *k* variables in Eq. (7) that lead to distinct $ChF_0$ is the multinomial coefficient $\frac{k!}{m_1!m_2!\ldots m_i! \ldots m_{last}!}$, where $m_i$ is the layer-size of the *i*th layer. The number of negations (including the identity operation) of a $ChF_0$ that lead to distinct $ChF_0$s is $1+m_{last}$. Now for each permutation there are $1+m_{last}$ possible negations, therefore the number of *k*-input $ChF_0$ for a given bias *P* is:

10 $$\begin{aligned} |ChF_0|_{k,P} = \frac{k! ~ (1+m_{last})}{m_1!m_2!\ldots m_{last}!} \end{aligned}$$

Using Eq. (10), the total number of *k*-input $ChF_0$ is given by,

11 $$\begin{aligned} |ChF_0|_{k} = \sum _{\begin{array}{c} 1 \le P < 2^{k-1},\\ P \text{ odd} \end{array}} \frac{2\cdot k! ~ (1+m_{last})}{m_1! m_2! \ldots m_{last}!} \end{aligned}$$

We verify that the number of *k*-input $ChF_0$ as given by the Eq. (11) matches with the numbers obtained by computationally enumerating the *k*-input $ChF_0$ functions (see https://github.com/asamallab/GenChF for code) and also the numbers provided by Gat-Viks and Shamir^26^ (Gat-Viks and Shamir provide values upto $k=6$). *P* is odd because $ChF_0$ have odd bias. Furthermore, the factor 2 in this equation accounts for a complementary function with bias $2^k - P$ associated with each $ChF_0$ with bias $P < 2^{k-1}$ since the layer-number and layer-size are invariant under complementation for any NCF. The total number of BFs belonging to the class $ChF_U$ for $k \ge 3$ is then given by:

12 $$\begin{aligned} |ChF_U|_{k} = |ChF_0|_{k} + |ChF_1|_{k} = \sum _{\begin{array}{c} 1 \le P < 2^{k-1},\\ P \text{ odd} \end{array}} \frac{4 \cdot k! ~ (1+m_{last})}{m_1! m_2! \ldots m_{last}!} \end{aligned}$$

since $|ChF_0|_{k} = |ChF_1|_{k}$ (see SI text, Property 2.2) and for $k \ge 3$, $ChF_0$ and $ChF_1$ form disjoint sets. Note, for $k \le 2$ (see SI text, Property 2.1),

$$\begin{aligned} |ChF_U|_{k} = |NCF|_{k} \end{aligned}$$

### The fraction of chain-0 and chain-1 within NCFs decreases exponentially with the number of inputs

The fraction of NCFs occupied by $ChF_0$ for different number of inputs is yet to be explored systematically. To begin, we compute the fraction of NCFs that are in $ChF_0$ for $k \in \{1,2,\ldots ,30\}$ using exhaustive enumeration. As expected, this fraction appears to diminish exponentially as *k* grows as shown by the linear trend in the semi-log plot in Fig. 2b. Furthermore, the generalized chain functions ($ChF_U$) also show this trend since the cardinality of that class of functions is a factor 2 larger than that of $ChF_0$ for all $k \ge 3$ (see SI text, Property 2.2). The fraction of NCFs belonging to a *k*-input $ChF_0$ can be written in the following form (see SI text, section 3 for derivation):

$$\begin{aligned} \frac{|ChF_0|_k}{|NCF|_k}= & {} \frac{1}{2^k} C \\ \text{where}~~ C= & {} \frac{\sum _{\begin{array}{c} 1 \le P< 2^{k-1},\\ P \text{ odd} \end{array}} \frac{1+m_{last}}{m_1! m_2! \ldots m_{last}!}}{\sum _{\begin{array}{c} 1 \le P < 2^{k - 1},\\ P \text{ odd} \end{array}} \frac{1}{m_1!m_2!\ldots m_{last}!}} \end{aligned}$$

Since $m_{last} \ge 2$ for all $k \ge 2$, one has $C \ge 3$. Since $m_{last}$ is at most *k* for any *k*, $C \le k+1$. From this it follows that the fraction $1/2^k$ accounts for the observed exponential decrease. Exhaustive computation up to $k = 30$ suggests that *C* converges at large *k* towards a value $\approx 3.2588913532709$. As the cardinality of $ChF_0$ is equal to that of $ChF_1$ (see SI text, Property 2.2), all the results obtained for $ChF_0$ are equally valid for $ChF_1$. Note that this exponential decrease has important implications for restricting the space of biologically plausible models as we will demonstrate in later sections.

**Figure 3:**
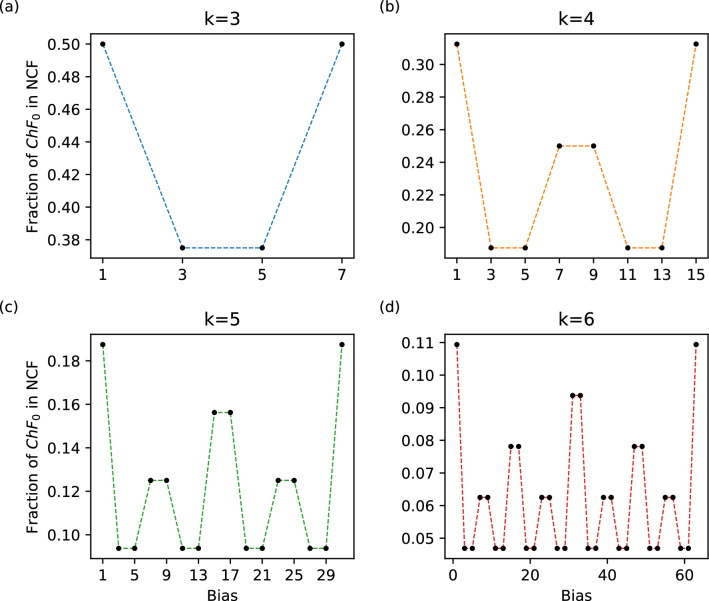
Bias-wise fraction of chain-0 (or chain-1) within NCFs. For a given number of inputs (*k*), the fraction of $ChF_0$ (or $ChF_1$) in NCFs (*y*-axis) is plotted as a function of the bias ($1\le P \le 2^{k}-1$ for odd *P*) (*x*-axis). Subplots correspond to different number of inputs $k=3,4,5$ and 6.

### Bias-wise fractions of chain-0 and chain-1 functions within NCFs

To study the behaviour of how the fractions of $ChF_0$ (or $ChF_1$) and NCFs within all BFs, and with respect to one another, vary with bias (*P*) for a given number of inputs (*k*), we plot the fraction of: (1) NCFs within all BFs (see SI Fig. S1); (2) $ChF_0$ within all BFs (see SI Fig. S2); and (3) $ChF_0$ within NCFs (see Fig. 3). Of these, the fraction $ChF_0$ within NCFs (see Fig. 3) showed several interesting features. Note that since the cardinality of $ChF_0$ is equal to that of $ChF_1$ (see SI text, Property 2.2) for a given *k* and *P*, all results obtained for $ChF_0$ are equally valid for $ChF_1$. We list below our observations and will explain them with the following simple and elegant formula derived using Eqs. (5) and (10):

13 $$\begin{aligned} f_{CN}(k,P) = \frac{|ChF_0|_{k,P}}{|NCF|_{k,P}} = \frac{|ChF_1|_{k,P}}{|NCF|_{k,P}} = \frac{1+m_{last}}{2^{k}}. \end{aligned}$$

The observations are as follows:

1. $f_{CN}(k,P) = f_{CN}(k,P+2)$ whenever $P= 4t + 3$ for any $t \in \mathbb{N}_{0}$. In other words, those particular pairs of consecutive biases (starting from $P=3$) have equal $f_{CN}(k,P)$. This implies that for any *k*, the $m_{last}$ for consecutive biases (see Eq. (13)) are equal. This is indeed the case as we prove in SI text, Property 1.3.
2. Since $m_{last}$ determines $f_{CN}(k,P)$, biases with equal $m_{last}$ also have equal $f_{CN}(k,P)$. For example, using SI text, Property 1.3 , $m_{last} = 2$ for $P=3,5,11,13,19,21 \ldots$. This explains why several biases in Fig. 3 have the same $f_{CN}(k,P)$.
3. $f_{CN}(k,P)$ is maximum when $P=1$ or $2^k-1$ for a given *k* and it is minimum when $P=3,5,11,13,19,21,\ldots$. For $k \ge 2$, we know that $2 \le m_{last} \le k$. So, $f_{CN}(k,P)$ is maximum (respectively minimum) when $m_{last} = k$ (respectively $m_{last} = 2$). $m_{last} = k$ iff $P=1$ or $P=2^k - 1$ since $m_{last} = k$ corresponds to a $ChF_0$ (or $ChF_1$) with a single layer. The maximum value of $f_{CN}(k,P)$ is then ${(1+k)}/{2^k}$. However $m_{last} = 2$ for several values of *P* (see SI text, Property 1.4). The minimum value of $f_{CN}(k,P)$ is ${(1+2)}/{2^k} = {3}/{2^k}$ (when $k \ge 2$) and ${(1+1)}/{2^1} = 1$ (when $k=1$).
4. $f_{CN}(k+1,P\ne 1) = \frac{1}{2} f_{CN}(k,P \ne 1)$ and $f_{CN}(k+1,P=1) = \frac{1}{2^{k+1}} + \frac{1}{2} f_{CN}(k,P=1)$. From Eq. (13), it is easy to see that $f_{CN}(k+1,P\ne 1) = \frac{1}{2} f_{CN}(k,P\ne 1)$ since $m_{last}$ for $P\ne 1$ remains invariant for any *k* (see SI text, property 1.2). However, when $P=1$, $m_{last}=k$ and so $f_{CN}(k,P=1) = {(1+k)}/{2^k}$. Note that the result *does* hold for $P=2^k-1$. Replacing *k* by $k+1$ in this equation, $f_{CN}(k+1,P=1) = \frac{1}{2^{k+1}} + \frac{1}{2} f_{CN}(k,P=1)$.

### Preponderance and enrichment of chain-0, chain-1 and generalized chain functions in various reference biological datasets

We now shift our focus to quantifying the preponderance of $ChF_0$, $ChF_1$ and $ChF_U$ in various biological datasets of regulatory logic rules.

#### BBM benchmark dataset

**Figure 4:**
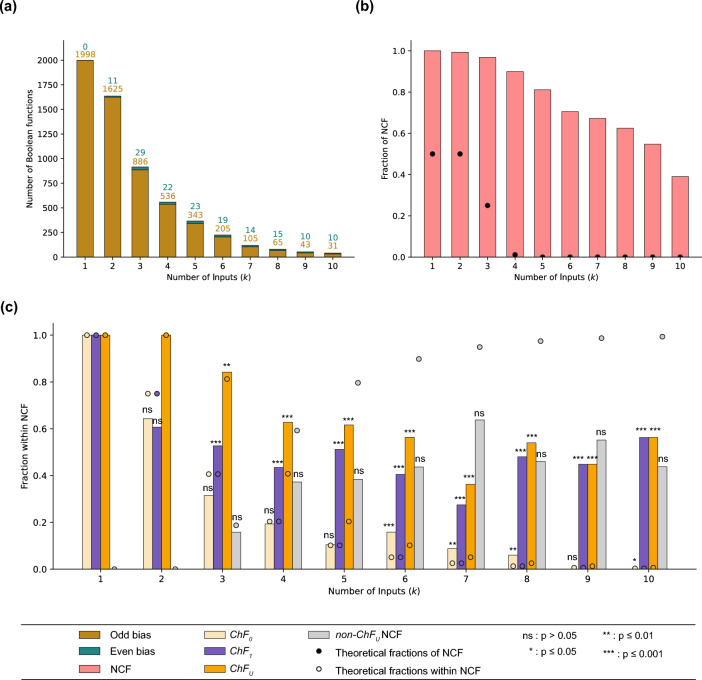
Fractions of various sub-types within NCFs in the BBM benchmark dataset. **(a)** In-degree distribution of BFs in the BBM benchmark dataset up to $k \le 10$ inputs. The frequencies of odd bias are much larger than the frequencies of even bias. **(b)** The fraction of NCFs in the dataset for various number of inputs. The enrichment of NCFs (within all BFs) for any number of inputs is very large and also statistically significant in the BBM benchmark dataset. **(c)** This sub-figure shows the fractions of $ChF_0$, $ChF_1$, $ChF_U$ and *non*-$ChF_U$ NCF within NCFs, in theory and in the BBM benchmark dataset as dots and colored bars respectively. The relative enrichments of $ChF_1$s and $ChF_U$s within NCFs are statistically significant for $k>2$.

We first consider the case of the BBM benchmark dataset^29^. When we quantify the fraction of odd and even bias BFs for a given number of inputs (*k*), we find that the fraction of BFs with odd bias are overwhelmingly larger compared to the fraction of BFs with even bias (see Fig. 4a). Next, we compute the fraction of NCFs for each *k* and find that it is enriched for all *k* as shown in Fig. 4b. Both of these observations are in line with previous studies on the fraction of odd bias BFs and the fraction of NCFs in reference biological datasets^13^. Lastly, for a *k*-input BF belonging to the sub-types of NCFs, namely, $ChF_0$, $ChF_1$, $ChF_U$ and *non*-$ChF_U$ NCF, we compute their fraction within the NCFs (shown by the bars in Fig. 4c and SI Table S6), their relative enrichments within the NCFs (see SI Table S7) and the associated statistical significance of those enrichments (see $*$ in Fig. 4c and SI Table S8 for exact values). We find that $ChF_1$ is relatively enriched for all values of *k* except at $k=2$. However, this is not the case for $ChF_0$ which is not enriched for several values of *k* ($k=3,4,5$ and 9). The union of these two types, $ChF_U$, is significantly enriched for all $k > 2$. These results suggest that the generalized chain function, $ChF_U$, consisting of both $ChF_0$ and $ChF_1$ types perhaps constitutes a biologically more meaningful type than either the $ChF_0$ or $ChF_1$ separately. Note that at $k\le 2$ since all NCFs are also generalized chain functions, it is meaningless to compute a relative enrichment or statistical significance.

#### MCBF and Harris datasets

Repeating the above-mentioned set of analyses for the MCBF dataset, we find that for a given number of inputs, the fraction of the $ChF_1$ type within NCFs is typically larger than that of the $ChF_0$ and *non*-$ChF_U$ NCF types within the NCFs (see SI Fig. S3(a) and SI Table S9). In fact, $ChF_1$ is relatively enriched within NCFs for all values of *k* except $k=2$, whereas $ChF_0$ is relatively enriched only when $k=6,7,8$ (see SI Fig. S3(a) and SI Table S10). Furthermore, $ChF_U$ is relatively enriched within NCFs for all $k \ge 3$, but only those enrichments where $k>3$ are statistically significant (see **Methods**). The complete set of values for the statistical significance associated with the relative enrichments is provided in SI Table S11. These results obtained using the MCBF dataset are largely in agreement with those of the BBM benchmark dataset.

We now revisit an older dataset of BFs, namely the one published by Harris *et al.*^3,27^. In this dataset, we find that for any given number of inputs, the fraction of $ChF_0$ within NCFs is larger than the corresponding fractions of $ChF_1$ and *non*-$ChF_U$ NCF types (see SI Fig. S3(b) and SI Table S12). Furthermore, the $ChF_0$ BFs are relatively enriched within NCFs and the enrichments are statistically significant (see SI Tables S13 and S14 respectively). $ChF_1$ on the other hand is not relatively enriched within NCFs. The generalized chain functions, $ChF_U$, are enriched for $k=3,4,5$ but those enrichments are statistically significant only for $k=4,5$. These results are not so concordant with those arising from the BBM benchmark dataset in which the relative enrichment of $ChF_0$ is not statistically significant for $k=3,4,5$, whereas it is so for $ChF_1$. It is appropriate to note here that Kauffman *et al.*^3^ had observed that a significant fraction of the BFs in that dataset were NCFs and that most of those NCFs were $ChF_0$.

In sum, the generalized chain functions (or $ChF_U$) comprise a special sub-type of NCFs that are quite consistently relatively enriched within NCFs across distinct reference biological datasets and are thereby able to reconcile the discrepancies observed in the relative enrichments of $ChF_0$ and $ChF_1$ within NCFs therein.

### Model selection using generalized chain functions

In this section, we explore the implications of using generalized chain functions as a constraint in a model selection framework. To do so, we consider 3 biological models, one Pancreas cell differentiation model and two *Arabidopsis thaliana* root development models (see **Methods**).

**Table 1:** Nodewise enumeration of the number of BFs that satisfy biological constraints for the RSCN-2010 GRN.

Nodes	*k*	$ChF_0$	$ChF_1$	$ChF_U$
PLT	1	1	1	1
AUXIN	1	1	1	1
ARF	1	1	1	1
AUXIAA	1	1	1	1
SHR	1	1	1	1
SCR	4	5	0	5
JKD	2	1	0	1
MGP	3	1	0	1
WOX5	5	12	0	12

‘Nodes’ are the names of the nodes in the network and *k* is the associated number of inputs to that node. The columns $ChF_0$, $ChF_1$ and $ChF_U$ give the number of chain-0, chain-1 and generalized chain functions that satisfy biological fixed point constraints and sign conforming constraints at each node of the RSCN-2010 GRN. Imposing $ChF_U$ leads to 60 Boolean models.

First, we apply the framework described in **Methods** using the generalized chain functions ($ChF_U$) as our biologically meaningful type of BF (as opposed to the generally utilized NCF). The number of allowed BFs at each node of these models (pancreas cell differentiation, RSCN-2010, RSCN-2020) are given in SI Table S15, Table 1 and SI Table S16 respectively. The numbers of models obtained for these three cases are $3600,\ 60$ and 645120 respectively. We remark here that in the RSCN-2020 GRN, no $ChF_U$ satisfied both fixed point and sign conforming constraints at the ARF10 node and so for that node we restricted our choice to NCFs, leading to 4 BFs at ARF10. Note that if we restrict our choice of BFs to NCFs for all nodes in all 3 biological models, we would have got 3600, 1275 and 25019245440 $(2.5 \times 10^{10})$. Clearly, the resulting number of biologically plausible models is severely reduced (particularly for models having nodes with large number of inputs) compared to the case where NCFs are used as a constraint. This allows us to *exhaustively* search these ensembles for models that satisfy the relative stability constraints provided in **Methods**.

Next, we impose known relative stability constraints (see **Methods**) to shrink the space of biologically plausible models further. We utilized the synchronous update scheme to check which of the models satisfied the relative stability constraints for model selection. Using the MFPT as our relative stability measure, we find that: (1) 19 models satisfy the expected hierarchies (which is the stability hierarchies of the fixed point attractors) for the pancreas cell differentiation GRN with no spurious attractors. (2) 16 models satisfy the expected hierarchies for the RSCN-2010 GRN with no spurious attractors. (3) For the RSCN-2020 GRN, no model satisfied all the expected hierarchies. However, we found that 82355 models violated exactly one hierarchy, namely, ‘C.ProvascularPD < C.ProvascularTD2’ of which 28453 did not have any spurious attractors. This result is still a major improvement over the original Boolean model proposed by García-Gómez *et al.*^33^ which violated 2 expected hierarchies (‘C.ProvascularPD < C.ProvascularTD2’ and ‘QC < CEI/EndodermisPD’) and furthermore had a spurious cyclic attractor. In all, for the RSCN-2020 GRN, about $4.4\%$ of models that formed our biologically plausible ensemble using $ChF_U$ yielded improved models.

We have thus demonstrated the utility of generalized chain functions as a biologically meaningful type that can severely restrict the space of biologically plausible models and yet can yield models that satisfy conditions on relative stability.

## Discussion and conclusions

In this work, we have addressed the question of whether there are certain sub-types of NCFs that drive the enrichment of the NCFs in reconstructed Boolean models of biological networks. Starting with a known sub-type of NCF, namely the chain functions (or chain-0 functions), we propose two other types, specifically, its dual class—the chain-1 functions, and its union with the chain-1 functions, the generalized chain functions. We first derive an analytical formula to count these functions for a given number of inputs and a given bias. Using this we show that the fraction of chain-0 (or chain-1) functions decreases exponentially within NCFs as the number of inputs increases. Furthermore, our formula can explain several features of the pattern observed between the fraction of chain-0 (or chain-1) functions in NCFs and the bias, for a fixed number of inputs. We then test for enrichment of the chain-0, chain-1 and generalized chain function within NCFs in a large dataset of reconstructed Boolean models (the BBM benchmark dataset): the result is that generalized chain functions are indeed highly enriched. In fact, using 2 other datasets of regulatory logics, namely, the MCBF and the Harris dataset, the same result holds. In addition, we demonstrate how generalized chain functions can severely constrain the space of biologically plausible models using 3 different biological models. Lastly, we perform model selection on those models using known relative stability constraints and are able to zero in on a smaller subset of models that are more biologically plausible.

Since their introduction by Gat-Viks and Shamir^26^, the chain-0 functions have hardly received any attention. Kauffman *et al.*^3^ identified that chain-0 functions were the sub-type of NCFs for which all the canalyzing input values are 0 and those authors showed its preponderance in the dataset of Harris *et al.*^27^. Akutsu *et al.*^28^ put forward the Boolean expression framework associated with chain-0 functions^28^, making explicit the dependence of logical operators ($\wedge$, $\vee$) on the signs of the variables preceding it. Surprisingly, its dual class had never been proposed nor explored as a potentially biologically relevant type. Our work builds on all these works, piecing together several concepts pertaining to various representation of chain functions in a systematic manner, thereby providing insights into the nature of chain-0 (and chain-1) functions and the space they occupy within NCFs. Why are generalized chain functions preponderant in biological datasets? For one, the justifications for an enrichment of chain-0 functions given by Gat-Viks and Shamir^26^ also apply to chain-1 functions. Furthermore, we may speculate that a somewhat qualitative justification lies in that generalized chain functions have lower complexity than general NCFs when using the framework of Kauffman *et al.*, because of the very simple dependence of the operator following a variable and its sign (except for the last variable). The caveat here is that having that simpler description is dependent on the way one represents the functions, so for instance when using the representations of Gat-Viks and Shamir or of Akutsu *et al.*, the aforementioned simplicity is no longer apparent. One may also speculate that rather than being subject to a direct selection (e.g., for simplicity), chain functions are selected for indirectly, likely through their possible modulation of network dynamics. Indeed, network dynamics are subject to strong selection pressures, be-it for robustness or evolvability. It is also likely that the enrichment of chain functions is due to evolutionary constraints, for instance, selection for logic rules that lower or minimize protein production costs. Therefore, our results should have implications for understanding how molecular logic rules are shaped by evolutionary forces.

Although these results provide novel insights, it is appropriate to make explicit some limitations in this work. Firstly, we have seen that chain-0 functions were enriched in the Harris dataset, however, when considering much larger datasets such as the BBM benchmark dataset and MCBF dataset, there are multiple values of the number of inputs where there is no such enrichment. Similarly, the chain-1 functions are enriched in the BBM benchmark dataset and MCBF dataset, whereas they are not enriched in the Harris dataset. With more data, we may expect that several other sub-types of NCFs may also contribute to the enrichment of NCFs. Furthermore, there is likely to be an (un)conscious subjective bias on BF logic when reconstructing Boolean models, and so we cannot exclude that such a bias is responsible for the enrichments found. Secondly, the generalized chain functions can be so severely restrictive that it may be impossible to find corresponding BFs that satisfy both the fixed point and sign-conforming constraints as we found in the RSCN-2020 model. Lastly, relying *only* on “biologically meaningful” BFs can sometimes be too stringent. Cases wherein activatory or inhibitory regulation of a gene by a transcription factor may depend on the presence or absence of other transcription factors do exist—auxin response factors serve as good examples^43^. In such scenarios, it might be better to take a probabilistic approach to selecting BFs in the model selection framework, allowing for such exceptions with a low probability. In a broader perspective, automated methods of reconstruction, for still a long time, will require the intervention of biologists to check whether the set of models that are output are faithful to biological knowledge.

Our findings also invite several directions for future work. One is to quantify properties of the *generalized chain functions* that distinguish them from other NCFs and are likely responsible for their being so prevalent in these biological reference datasets. Another is to analytically tackle the question of whether the ratio of the number of the chain functions to that of the NCFs for a large number of inputs follows a rigorous exponential behaviour.

### Supplementary Information

Supplementary Information.

## Data Availability

All data and codes needed to reproduce the results in this manuscript are deposited in GitHub and are available at: https://github.com/asamallab/GenChF.
