# Supplementary material for: Preponderance of generalized chain functions in reconstructed Boolean models of biological networks
Source: Sci Rep. 2024 Mar 20;14:6734. doi: 10.1038/s41598-024-57086-y (PMC10954731; doi:10.1038/s41598-024-57086-y)
Supplement: Supplementary file 1 — Supplementary Information. [file 41598_2024_57086_MOESM1_ESM.pdf]

# SUPPLEMENTARY INFORMATION

## FOR

## Preponderance of generalized chain functions in reconstructed Boolean models of biological networks

**Suchetana Mitra[1,2,6], Priyotosh Sil[2,3,6], Ajay Subbaroyan[2,3,6], Olivier C. Martin[4,5,*], and Areejit Samal[2,3,*]**

[1]Indian Institute of Science Education and Research (IISER) Mohali, Punjab, India
[2]The Institute of Mathematical Sciences (IMSc), Chennai 600113, India
[3]Homi Bhabha National Institute (HBNI), Mumbai 400094, India
[4]Université Paris-Saclay, CNRS, INRAE, Univ Evry, Institute of Plant Sciences Paris-Saclay (IPS2), 91405 Orsay, France
[5]Université Paris-Cité, CNRS, INRAE, Institute of Plant Sciences Paris-Saclay (IPS2), 91405 Orsay, France
[6]S.M., P.S. and Aj.S. contributed equally to this work and should be considered as Joint-First authors
[*]To whom correspondence should be addressed: olivier.c.martin@inrae.fr; asamal@imsc.res.in

## 1 Relationship between bias, logical operators and layers in NCFs

**Property 1.1.** For any $k$-input NCF, the bias $P$ determines the operator sequence in its Boolean expression.

*Proof.* It is evident from the definition of NCF (see Main text, Definition 1) that the value of $b_i$ determines the operator following $x_{\sigma(i)}$ for $i \leq k-1$. Put simply, if $b_i$ is 0 or 1, the operator following $x_{\sigma(i)}$ should be $\wedge$ or $\vee$ respectively. So, there is a clear relation between the sequence of $b_i$ and the sequence of operators. Now, the value $b_i$ fills up $2^{k-i}$ rows of the truth table of the NCF. Hence, the bias ($P$) of a NCF can be written in the following form,

$$\begin{aligned} P &= b_1 2^{k-1} + b_2 2^{k-2} + \ldots + b_{k-1} 2^1 + b_k + \overline{b}_k \\ &= \sum_{i=1}^{k-1} b_i 2^{k-i} + 1 \end{aligned}$$

Thus the binary representation of the bias $P$ is given by $P_{Bin} = b_1 b_2 \ldots b_{k-1} 1$. This proves that from the binary representation of $P$ we can obtain the operator sequence of a NCF with bias $P$ such that the bits 0 and 1 encode the operators $\wedge$ and $\vee$ respectively while considering the sequence from left to right. □

**Property 1.2.** For all odd $P$ ($P \neq 1$), $m_{last}$ is independent of $k$ for any $k$-input NCF.

*Proof.* We can obtain the $(k+1)$-bit binary representation of a $(k+1)$-input NCF with bias $P$ by adding a 0 to the most significant end of the $k$-bit binary representation of bias $P$. More succintly, $P_{Bin}(k+1) =$ '$0P_{Bin}(k)$'. This does not alter the value of $m_{last}$ for all possible bias values except $P = 1$. When $P = 1$, adding a 0 at the most significant end increases the $m_{last}$ by 1 since $P = 1$ corresponds to a single layer NCF where all but the least significant bit is 0. □

**Property 1.3.** The values of $m_{last}$ for $k$-input NCFs with bias $P = 4t+3$ and bias $P' = 4t+5$ are equal, for any $t \in \mathbb{N}_0$.

*Proof.* We want to prove that, the values of the $m_{last}$ for the biases $3, 7, 11, 15, 19, \ldots$ will be equal to the values of the $m_{last}$ for the biases $5, 9, 13, 17, 21, \ldots$ respectively for any $k$-input NCF. Since the biases of the first category are of the form $4t+3$ for $t \in \mathbb{N}_0$, it becomes evident that the binary representations of these biases will have 1 as the bit to the left of the least significant bit. Consequently, it becomes apparent that their last layer is composed of 1s (or equivalently of '$\vee$' operators). Therefore, their

general binary form can be expressed as:

$$P_{Bin} = \ldots 0 \underbrace{\underbrace{111\ldots 11}_{m_{last}}}_{k}$$

Now, the next odd bias (each of which is of the form $4t+5$ for $t \in \mathbb{N}_0$) is obtained by adding 2 to $P$, which is the same as performing the Boolean addition $P_{Bin} + 00\ldots 0010$. This yields the following result:

$$\ldots 1 \underbrace{\underbrace{000\ldots 01}_{m_{last}}}_{k}$$

Hence, the value of $m_{last}$ will remain the same for a bias $P = 4t+3$ and $P' = 4t+5$, for any given value of $t \in \mathbb{N}_0$. □

**Property 1.4.** All bias $P$ ($\neq 1$) of a $k$-input NCF with $m_{last} = m$ can be expressed as $P = S \cdot 2^{m+1} + 2^m \pm 1$ for some $S \in \mathbb{N}_0$.

*Proof.* It is easy to see that the smallest possible value of $P$ for which we get $m_{last} = m$ for any $m$ will have the following binary representation

$$P_{Bin} = 0\ldots 00\underbrace{11\ldots 1}_{m}$$

So, the integer form of the bias $P$ is given by

$$P = 2^{m-1} + 2^{m-2} + \ldots + 2^2 + 2 + 1 = 2^m - 1 \quad (1)$$

Now, for $m = 2$ (the least possible value for $m$ is 2), $P = 2^2 - 1 = 3 = 4t+3$ where $t = 0$.
Again, for $m \geq 3$, $P$ can be expressed as

$$P = 2^2(2^{m-3} + \ldots + 2^1 + 1) + 3 \quad (2)$$
$$= 4t + 3 \quad (3)$$

where, $t = 2^{m-3} + \ldots + 2^1 + 1$. So, from Property 1.3, for the bias $P+2 = 2^m + 1$ also, the value of $m_{last} = m$. So, given $m_{last} = m$, the associated two lowest values of $P$ have the binary form

$$\bar{b}\underbrace{bb\ldots b}_{m-1}1$$

where $b \in \{0,1\}$ which takes the form $2^m + 1$ and $2^m - 1$ (when converted to the integer form) upon taking $b$ as 0 and 1 respectively. Now given the binary form $\bar{b}\underbrace{bb\ldots b}_{m-1}1$ one is free to add any number of bits to the left and $m_{last}$ will still remain equal to $m$. In other words, any bias $P'$ with the binary representation $a_n a_{n-1}\ldots a_1\bar{b}\underbrace{bb\ldots b}_{m-1}1$, $a_i \in \{0,1\}$ will have $m_{last} = m$, $\forall n \in \mathbb{N}$. We now express $P'$ in its integer form as follows:

$$\begin{aligned} P' &= a_n 2^{n+m} + a_{n-1}2^{n+m-1}\ldots a_1 2^{m+1} + (2^m \pm 1) \\ &= 2^{m+1}(a_n 2^{n-1} + a_{n-1}2^{n-2} + \ldots + a_1) + 2^m \pm 1 \\ &= 2^{m+1} \cdot S + 2^m \pm 1 \end{aligned}$$

where $S \in \mathbb{N}$ can be expressed in the form $a_n 2^{n-1} + a_{n-1}2^{n-2} + \ldots + a_1$ with suitable choices of $a_i$'s and $n$. □

## 2 Properties of chain-0 and chain-1 functions

**Property 2.1.** For $k \geq 3$, the two sub-types of NCF namely $ChF_0$ and $ChF_1$ forms disjoint classes.

*Proof.* Let us discuss the scenario for different values of $k$.

- $\boldsymbol{k = 1}$ **:** There are only 2 NCFs for $k = 1$ and those are $x_1$ and $\bar{x}_1$. They are both $ChF_0$ and $ChF_1$. So, for $k = 1$ the three classes NCF, $ChF_0$ and $ChF_1$ are identical:

$$ChF_U = ChF_0 \cup ChF_1 = ChF_0 \cap ChF_1 = \{x_1, \bar{x}_1\} = \text{NCF}$$

- **$k = 2$ :** There are 8 NCFs for $k = 2$. From the definitions of $ChF_0$ (see Main text, **Methods** section) and $ChF_1$ (see Main text, **Methods** section), the 6 $ChF_0$s are $x_1 \wedge x_2$, $x_1 \wedge \bar{x}_2$, $\bar{x}_1 \wedge x_2$, $\bar{x}_1 \vee \bar{x}_2$, $x_1 \vee \bar{x}_2$ and $\bar{x}_1 \vee x_2$ and the 6 $ChF_1$s are $x_1 \vee x_2$, $x_1 \vee \bar{x}_2$, $\bar{x}_1 \vee x_2$, $\bar{x}_1 \wedge \bar{x}_2$, $x_1 \wedge \bar{x}_2$ and $\bar{x}_1 \wedge x_2$. Hence we see that,

$$ChF_0 \cap ChF_1 = \{x_1 \wedge \bar{x}_2, \bar{x}_1 \wedge x_2, x_1 \vee \bar{x}_2, \bar{x}_1 \vee x_2\} \neq \phi.$$

  Again,

$$ChF_0 \cup ChF_1 = ChF_U = \{x_1 \wedge x_2, x_1 \wedge \bar{x}_2, \bar{x}_1 \wedge x_2, \bar{x}_1 \vee \bar{x}_2, x_1 \vee \bar{x}_2, \bar{x}_1 \vee x_2, x_1 \vee x_2, \bar{x}_1 \wedge \bar{x}_2\} = \text{NCF}$$

  Hence, for $k = 2$, the classes $ChF_0$ and $ChF_1$ are not disjoint.

- **$k \geq 3$ :** Clearly, a multi-layer NCF can never simultaneously be a $ChF_0$ and $ChF_1$ since for each there is a strict dependency between the variable signs and the operators ($\wedge$ and $\vee$) within all but the last layer and no function can satisfy these distinct dependencies at once. So, let us consider a $k$-input ($k \geq 3$) $ChF_0$, $f$ with single layer. Then, $m_{last} = k$. Now, $f$ can have at most one inconsistency with the variable sign and the operator. Subsequently it follows that $f$ will have at least $(k-1)$ inconsistencies between the variable signs and the operators required to get a $ChF_1$. Since $k \geq 3$, $(k-1) \geq 2$. This violates the sign-operator relationship for the $ChF_1$ expression and hence $f$ cannot be a $ChF_1$. By symmetry, a $ChF_1$ for $k \geq 3$ cannot be a $ChF_0$. Hence, for $k \geq 3$,

$$ChF_0 \cap ChF_1 = \phi$$

  For all $k \geq 3$ there exists multi-layer NCFs, some of which will always violate the condition of $ChF_0$ (similarly $ChF_1$) at least at one layer. Hence, for $k \geq 3$,

$$ChF_U \neq \text{NCF}$$

  As an example, consider the 3-input NCF, $g = x_1 \vee (x_2 \wedge x_3)$. Then, $g$ violates the condition of being $ChF_0$ in the first layer and the condition to be $ChF_1$ in the second (last) layer and hence, this NCF is neither a $ChF_0$ nor a $ChF_1$.

□

**Property 2.2.** The cardinality of $k$-input $ChF_0$ or $ChF_1$ with bias $P$ are equal. As a result, the number of $k$-input $ChF_0$ and $k$-input $ChF_1$ is equal for any $k$.

*Proof.* Let us consider a map $f_p$ whose domain is the class of $k$-input $ChF_0$ with bias $P$ and co-domain is $k$-input $ChF_1$ with bias $P$. The map is defined by its flipping the signs of all the variables. Clearly, $f_p$ is a one-to-one map. Hence, $|ChF_0|_{k,P} \leq |ChF_1|_{k,P}$ where, $|ChF_0|_{k,P}$ and $|ChF_1|_{k,P}$ correspond to the cardinality of the classes of $k$-input $ChF_0$ and $ChF_1$ with bias $P$ respectively. In exactly similar manner one can define a one-to-one map from the class of $ChF_1$ to the class of $ChF_0$. Then, $|ChF_1|_{k,P} \leq |ChF_0|_{k,P}$. This proves that, $|ChF_0|_{k,P} = |ChF_1|_{k,P}$. Hence, the total number of $k$-input $ChF_0$ functions is equal to the total number of $k$-input $ChF_1$ since,

$$|ChF_0|_k = \sum_{\substack{1 \leq P \leq 2^k, \\ P \text{ odd}}} |ChF_0|_{k,P} = \sum_{\substack{1 \leq P \leq 2^k, \\ P \text{ odd}}} |ChF_1|_{k,P} = |ChF_1|_k$$

□

## 3 The fraction of chain-0 (or chain-1) within NCFs decreases exponentially with number of inputs

We provide here the derivation of the fraction of $k$-input $ChF_0$ in NCFs for a given number of inputs $k$. The number of $k$-input NCFs is given by (see Main text, equation (5)),

$$|NCF|_k = \sum_{\substack{1 \leq P < 2^{k-1}, \\ P \text{ odd}}} \frac{2^{k+1} \cdot k!}{m_1! m_2! \ldots m_{last}!}$$

Again, the number of $k$-input $ChF_0$ function is given by (see Main text, equation (11)),

$$|ChF_0|_k = \sum_{\substack{1 \leq P < 2^{k-1}, \\ P \text{ odd}}} \frac{2 \cdot k! \, (1 + m_{last})}{m_1! m_2! \ldots m_{last}!}$$

Hence, the fraction of $ChF_0$ within NCFs for some given value of $k$ is,

$$\frac{|ChF_0|_k}{|NCF|_k} = \frac{\sum_{\substack{1\le P<2^{k-1},\\ P \text{ odd}}} \frac{2\ k!\ (1+m_{last})}{m_1!m_2!...m_{last}!}}{\sum_{\substack{1\le P<2^{k-1},\\ P \text{ odd}}} \frac{2^{k+1}k!}{m_1!m_2!...m_{last}!}} = \frac{1}{2^k}C \tag{4}$$

where,

$$C = \frac{\sum_{\substack{1\le P<2^{k-1},\\ P \text{ odd}}} \frac{1+m_{last}}{m_1!m_2!...m_{last}!}}{\sum_{\substack{1\le P<2^{k-1},\\ P \text{ odd}}} \frac{1}{m_1!m_2!...m_{last}!}}$$

.

## Supplementary Tables

**Table S1. Biological fixed points of the Pancreas cell differentiation model.** The 3 biological fixed points of the Pancreas cell differentiation model are the Exocrine, $\beta/\delta$ cell progenitor and $\alpha$/PP cell progenitor.

| Cell types | Nodes | | | | |
|---|---|---|---|---|---|
| | Pdx1 | Ptf1a | Ngn3 | Pax4 | Arx |
| Exocrine | 0 | 1 | 0 | 0 | 0 |
| $\beta/\delta$ cell progenitor | 1 | 0 | 1 | 1 | 0 |
| $\alpha$/PP cell progenitor | 0 | 0 | 1 | 0 | 1 |

**Table S2. Boolean functions of the published RSCN-2010 model in BoolNet format.** The column with header 'Target node name' contains the list of nodes whose regulation is captured by the corresponding row entry in the column 'Regulatory logic rule'. The symbols &, | and ! correspond to the logic operators AND, OR and NOT respectively.

| Serial Number | Target node name | Regulatory logic rule |
|---|---|---|
| 1 | SCR | ( SHR & SCR & !JKD & !MGP ) \| ( SHR & SCR & JKD & !MGP ) \| ( SHR & SCR & JKD & MGP ) |
| 2 | PLT | ARF |
| 3 | ARF | !AUXIAA |
| 4 | AUXIAA | !AUX |
| 5 | AUX | !AUX \| AUX |
| 6 | SHR | SHR |
| 7 | JKD | SHR & SCR |
| 8 | MGP | SHR & SCR & !WOX5 |
| 9 | WOX5 | (ARF & SHR & SCR & !MGP & !WOX5 ) \| (ARF & SHR & SCR & !MGP & WOX5 ) \| ( ARF & SHR & SCR & MGP & WOX5 ) |

**Table S3. Biological fixed points recovered by the RSCN-2010 model.** The 4 biological fixed points recovered by the model are the Quiescent center (QC), Vascular initials (VI), Cortex-Endodermis initials (CEI) and Columella epidermis initials (CEpI).

| **Cell types** | **Nodes** | | | | | | | | |
|---|---|---|---|---|---|---|---|---|---|
| | SCR | PLT | ARF | AUXIAA | AUXIN | SHR | JKD | MGP | WOX5 |
| QC | 1 | 1 | 1 | 0 | 1 | 1 | 1 | 0 | 1 |
| VI | 0 | 1 | 1 | 0 | 1 | 1 | 0 | 0 | 0 |
| CEI | 1 | 1 | 1 | 0 | 1 | 1 | 1 | 1 | 0 |
| CEpI | 0 | 1 | 1 | 0 | 1 | 0 | 0 | 0 | 0 |

**Table S4. Boolean functions of the published RSCN-2020 model in BoolNet format.** The column with header 'Target node name' contains the list of nodes whose regulation is captured by the corresponding row entry in the column 'Regulatory logic rule'. The symbols &, | and ! correspond to the logic operators AND, OR and NOT respectively.

| **Serial Number** | **Target node name** | **Regulatory logic rule** |
|---|---|---|
| 1 | CK | (PHB & !ARF) \| !SHR |
| 2 | ARR1 | !SCR & CK |
| 3 | SHY2 | ARR1 & !AUX |
| 4 | AUXIAA | !AUX |
| 5 | ARF | !AUXIAA |
| 6 | ARF10 | !(JKD & SHR) & !AUXIAA |
| 7 | ARF5 | ((PHB \| PLT) & !(SHR & MGP)) & !SHY2 & !AUXIAA |
| 8 | XAL1 | ARF |
| 9 | PLT | ARF5 \| ARF \| WOX5 \| XAL1 |
| 10 | AUX | AUX |
| 11 | SCR | SHR & JKD & SCR |
| 12 | SHR | SHR \| (SCR & JKD) |
| 13 | MIR166 | (SCR & SHR & !ARR1) \| !PHB |
| 14 | PHB | ((!ARR1 & PLT) \| PHB) & !MIR166 |
| 15 | JKD | !PHB & SHR & SCR |
| 16 | MGP | !ARF5 & SHR & SCR & MGP |
| 17 | WOX5 | !ARF10 & ARF5 & !CLE40 & SCR & PLT |
| 18 | CLE40 | !SHR |

**Table S5. Biological fixed points recovered by the RSCN-2020 model.** The 6 biological fixed points recovered by the model are the Quiescent center (QC), Cortex/endodermis initial cell (CEI/ EndodermisPD), Peripheral Pro-vascular initials (P.ProvascularPD), Central Pro-vascular initials (C.ProvascularPD), Transition domain (C.ProvascularTD2) and Columella initials (Columella1).

| Cell types | Nodes | | | | | | | | | | | | | | | | | |
|---|---|---|---|---|---|---|---|---|---|---|---|---|---|---|---|---|---|---|
| | CK | ARR1 | SHY2 | AUXIAA | ARF | ARF10 | ARF5 | XAL1 | PLT | AUX | SCR | SHR | MIR166 | PHB | JKD | MGP | WOX5 | CLE40 |
| QC | 0 | 0 | 0 | 0 | 1 | 0 | 1 | 1 | 1 | 1 | 1 | 1 | 1 | 0 | 1 | 0 | 1 | 0 |
| CEI/EndodermisPD | 0 | 0 | 0 | 0 | 1 | 0 | 0 | 1 | 1 | 1 | 1 | 1 | 1 | 0 | 1 | 1 | 0 | 0 |
| P.ProvascularPD | 0 | 0 | 0 | 0 | 1 | 1 | 1 | 1 | 1 | 1 | 0 | 1 | 1 | 0 | 0 | 0 | 0 | 0 |
| C.ProvascularPD | 0 | 0 | 0 | 0 | 1 | 1 | 1 | 1 | 1 | 1 | 0 | 1 | 0 | 1 | 0 | 0 | 0 | 0 |
| C.ProvascularTD2 | 1 | 1 | 0 | 0 | 1 | 1 | 1 | 1 | 1 | 1 | 0 | 0 | 0 | 1 | 0 | 0 | 0 | 1 |
| Columella1 | 1 | 1 | 0 | 0 | 1 | 1 | 1 | 1 | 1 | 1 | 0 | 0 | 1 | 0 | 0 | 0 | 0 | 1 |

**Table S6. Numbers and fractions of various sub-types of the NCFs in the BBM benchmark dataset.** $k$ is the number of inputs to a BF. The columns under the 'Number of BFs' grouping give the total number of BFs in the BBM benchmark dataset ('Total' column) and the numbers of BFs belonging to NCFs, $ChF_0$, $ChF_1$, $ChF_U$ and $non\text{-}ChF_U$ NCF types for various number of inputs. The columns under the 'Fraction of sub-types within NCFs' grouping give the fraction of the different sub-types of NCFs, namely, $ChF_0$, $ChF_1$, $ChF_U$ and $non\text{-}ChF_U$ NCF within the NCFs for various number of inputs.

| $k$ | Number of BFs | | | | | | Fraction of sub-types within NCFs | | | |
|---|---|---|---|---|---|---|---|---|---|---|
| | Total | NCF | $ChF_0$ | $ChF_1$ | $ChF_U$ | $non\text{-}ChF_U$ NCF | $ChF_0$ | $ChF_1$ | $ChF_U$ | $non\text{-}ChF_U$ NCF |
| 1 | 1998 | 1998 | 1998 | 1998 | 1998 | 0 | 1.0 | 1.0 | 1.0 | 0.0 |
| 2 | 1636 | 1625 | 1045 | 986 | 1625 | 0 | 0.64308 | 0.60677 | 1.0 | 0.0 |
| 3 | 915 | 886 | 279 | 467 | 746 | 140 | 0.3149 | 0.52709 | 0.84199 | 0.15801 |
| 4 | 558 | 502 | 97 | 218 | 315 | 187 | 0.19323 | 0.43426 | 0.62749 | 0.37251 |
| 5 | 366 | 297 | 31 | 152 | 183 | 114 | 0.10438 | 0.51178 | 0.61616 | 0.38384 |
| 6 | 224 | 158 | 25 | 64 | 89 | 69 | 0.15823 | 0.40506 | 0.56329 | 0.43671 |
| 7 | 119 | 80 | 7 | 22 | 29 | 51 | 0.0875 | 0.275 | 0.3625 | 0.6375 |
| 8 | 80 | 50 | 3 | 24 | 27 | 23 | 0.06 | 0.48 | 0.54 | 0.46 |
| 9 | 53 | 29 | 0 | 13 | 13 | 16 | 0.0 | 0.44828 | 0.44828 | 0.55172 |
| 10 | 41 | 16 | 0 | 9 | 9 | 7 | 0.0 | 0.5625 | 0.5625 | 0.4375 |

**Table S7. Relative enrichment ratios of various sub-types of NCFs within NCFs in the BBM benchmark dataset.** $k$ is the number of inputs to a BF. $ChF_0$, $ChF_1$, $ChF_U$ and *non-*$ChF_U$ NCF correspond to the chain-0, chain-1, generalized chain functions and NCFs that are not generalized chain functions respectively. The columns $ChF_0$, $ChF_1$, $ChF_U$ and *non-*$ChF_U$ NCF are the relative enrichment ratios (see Main text, **Methods** section) for the associated sub-types within NCFs in the BBM benchmark dataset. $E_R > 1$ indicates that there is an enrichment of the sub-type within NCFs. NA implies that there are no BFs of the type *non-*$ChF_U$ NCF in theory.

| $k$ | Relative enrichment ratios ($E_R$) of sub-types within NCFs | | | |
|---|---|---|---|---|
| | $ChF_0$ | $ChF_1$ | $ChF_U$ | *non-*$ChF_U$ NCF |
| 1 | 1.0 | 1.0 | 1.0 | NA |
| 2 | 0.85744 | 0.80903 | 1.0 | NA |
| 3 | 0.77513 | 1.29745 | 1.03629 | 0.84274 |
| 4 | 0.9481 | 2.13078 | 1.53944 | 0.62882 |
| 5 | 1.02486 | 5.02514 | 3.025 | 0.48202 |
| 6 | 3.10739 | 7.95491 | 5.53115 | 0.48623 |
| 7 | 3.43675 | 10.80123 | 7.11899 | 0.6717 |
| 8 | 4.71326 | 37.70606 | 21.20966 | 0.47202 |
| 9 | 0.0 | 70.42801 | 35.21401 | 0.55884 |
| 10 | 0.0 | 176.74722 | 88.37361 | 0.4403 |

**Table S8. Statistical significance of the relative enrichments ($E_R$) of various sub-types of NCFs within NCFs for BFs in the BBM benchmark dataset.** $k$ is the number of inputs to a BF. The columns $ChF_0$, $ChF_1$, $ChF_U$ and *non-*$ChF_U$ NCF give the $p$-values associated with the relative enrichment (see Main text, **Methods** section) of these sub-types of NCFs, within NCFs, for the BBM benchmark dataset. $p$-values $< 0.05$ are considered statistically significant. These $p$-values are used to assign the statistical significance stars in Fig. 4 in Main text. Note that all 2-input NCFs are $ChF_U$s, hence it is meaningless to compute $p$-values for both $ChF_U$ and *non-*$ChF_U$ NCF.

| $k$ | $p$-values associated with $E_R$ for sub-types of NCFs | | | |
|---|---|---|---|---|
| | $ChF_0$ | $ChF_1$ | $ChF_U$ | *non-*$ChF_U$ NCF |
| 2 | 1 | 1 | NA | NA |
| 3 | 1.00000 | $1.63713 \times 10^{-13}$ | 0.00982 | 0.98756 |
| 4 | 0.70020 | $5.94764 \times 10^{-32}$ | $1.13295 \times 10^{-23}$ | 1 |
| 5 | 0.39584 | $3.61377 \times 10^{-71}$ | $1.28372 \times 10^{-54}$ | 1 |
| 6 | $1.33886 \times 10^{-07}$ | $1.44303 \times 10^{-41}$ | $1.90778 \times 10^{-47}$ | 1 |
| 7 | 0.00100 | $3.61870 \times 10^{-18}$ | $1.14534 \times 10^{-18}$ | 1 |
| 8 | 0.00379 | $3.88012 \times 10^{-34}$ | $1.18719 \times 10^{-31}$ | 1 |
| 9 | 0.16904 | $1.27064 \times 10^{-23}$ | $1.90290 \times 10^{-19}$ | 1 |
| 10 | 0.04972 | $8.38830 \times 10^{-22}$ | $8.44118 \times 10^{-19}$ | 1 |

**Table S9. Numbers and fractions of various sub-types of NCFs in the MCBF dataset.** $k$ is the number of inputs to a BF. The columns under the 'Number of BFs' grouping give the total number of BFs in the MCBF dataset ('Total' column) and the numbers of BFs belonging to NCFs, $ChF_0$, $ChF_1$, $ChF_U$ and $non\text{-}ChF_U$ NCF types for various number of inputs. The columns under the 'Fraction of sub-types within NCFs' grouping give the fraction of the different sub-types of NCFs, namely, $ChF_0$, $ChF_1$, $ChF_U$ and $non\text{-}ChF_U$ NCF within the NCFs for various number of inputs.

| $k$ | Number of BFs | | | | | | Fraction of sub-types within NCFs | | | |
|---|---|---|---|---|---|---|---|---|---|---|
| | Total | NCF | $ChF_0$ | $ChF_1$ | $ChF_U$ | $non\text{-}ChF_U$ NCF | $ChF_0$ | $ChF_1$ | $ChF_U$ | $non\text{-}ChF_U$ NCF |
| 1 | 934 | 934 | 934 | 934 | 934 | 0 | 1.0 | 1.0 | 1.0 | 0.0 |
| 2 | 687 | 671 | 392 | 503 | 671 | 0 | 0.5842 | 0.74963 | 1.0 | 0.0 |
| 3 | 412 | 378 | 116 | 199 | 315 | 63 | 0.30688 | 0.52646 | 0.83333 | 0.16667 |
| 4 | 258 | 230 | 31 | 124 | 155 | 75 | 0.13478 | 0.53913 | 0.67391 | 0.32609 |
| 5 | 156 | 120 | 10 | 63 | 73 | 47 | 0.08333 | 0.525 | 0.60833 | 0.39167 |
| 6 | 107 | 67 | 10 | 26 | 36 | 31 | 0.14925 | 0.38806 | 0.53731 | 0.46269 |
| 7 | 51 | 34 | 4 | 10 | 14 | 20 | 0.11765 | 0.29412 | 0.41176 | 0.58824 |
| 8 | 45 | 27 | 2 | 9 | 11 | 16 | 0.07407 | 0.33333 | 0.40741 | 0.59259 |
| 9 | 19 | 7 | 0 | 2 | 2 | 5 | 0.0 | 0.28571 | 0.28571 | 0.71429 |
| 10 | 13 | 3 | 0 | 1 | 1 | 2 | 0.0 | 0.33333 | 0.33333 | 0.66667 |

**Table S10. Relative enrichment ratios of various sub-types of NCFs within NCFs in the MCBF dataset** $k$ is the number of inputs to a BF. $ChF_0$, $ChF_1$, $ChF_U$ and $non\text{-}ChF_U$ NCF correspond to the chain-0, chain-1, generalized chain functions and NCFs that are not generalized chain functions respectively. The columns $ChF_0$, $ChF_1$, $ChF_U$ and $non\text{-}ChF_U$ NCF are the relative enrichment ratios (see Main text, **Methods** section) for the associated sub-types within NCFs in the MCBF dataset. $E_R > 1$ indicates that there is an enrichment of the sub-type within NCFs. NA implies that there are no BFs of the type $non\text{-}ChF_U$ NCF in theory.

| $k$ | Relative enrichment ratios ($E_R$) of sub-types within NCFs | | | |
|---|---|---|---|---|
| | $ChF_0$ | $ChF_1$ | $ChF_U$ | $non\text{-}ChF_U$ NCF |
| 1 | 1.0 | 1.0 | 1.0 | NA |
| 2 | 0.77894 | 0.9995 | 1.0 | NA |
| 3 | 0.75539 | 1.29589 | 1.02564 | 0.88889 |
| 4 | 0.66133 | 2.64533 | 1.65333 | 0.55046 |
| 5 | 0.81824 | 5.1549 | 2.98657 | 0.49185 |
| 6 | 2.93115 | 7.62099 | 5.27607 | 0.51515 |
| 7 | 4.62085 | 11.55211 | 8.08648 | 0.6198 |
| 8 | 5.81884 | 26.18477 | 16.0018 | 0.60807 |
| 9 | 0.0 | 44.88818 | 22.44409 | 0.7235 |
| 10 | 0.0 | 104.7391 | 52.36955 | 0.67094 |

**Table S11. Statistical significance of the relative enrichments ($E_R$) of various sub-types of NCFs within NCFs for BFs in the MCBF dataset.** $k$ is the number of inputs to a BF. The columns $ChF_0$, $ChF_1$, $ChF_U$ and *non-*$ChF_U$ NCF give the $p$-values associated with the relative enrichment (see Main text, **Methods** section) of these sub-types of NCFs, within NCFs, for the MCBF dataset. $p$-values $< 0.05$ are considered statistically significant. These $p$-values are used to assign the statistical significance stars in Fig. S3. Note that all 2-input NCFs are $ChF_U$s, hence it is meaningless to compute $p$-values for both $ChF_U$ and *non-*$ChF_U$ NCF.

| $k$ | $p$-values associated with $E_R$ for sub-types of NCFs | | | |
|---|---|---|---|---|
| | $ChF_0$ | $ChF_1$ | $ChF_U$ | *non-*$ChF_U$ NCF |
| 2 | 1 | 0.49407518307496 | NA | NA |
| 3 | 0.99996 | $9.53875 \times 10^{-07}$ | 0.13412 | 0.83431 |
| 4 | 0.99561 | $8.77878 \times 10^{-30}$ | $1.08696 \times 10^{-16}$ | 1 |
| 5 | 0.68767 | $6.46209 \times 10^{-32}$ | $8.96155 \times 10^{-23}$ | 1 |
| 6 | 0.00054 | $6.69661 \times 10^{-18}$ | $8.58220 \times 10^{-20}$ | 1 |
| 7 | 0.00161 | $4.84767 \times 10^{-10}$ | $2.94472 \times 10^{-11}$ | 1.00000 |
| 8 | 0.00480 | $7.73712 \times 10^{-13}$ | $9.03023 \times 10^{-13}$ | 1.00000 |
| 9 | 0.04371 | $8.85440 \times 10^{-06}$ | $6.94878 \times 10^{-05}$ | 0.99674 |
| 10 | 0.00952 | $3.03207 \times 10^{-05}$ | 0.00012 | 0.98103 |

**Table S12. Numbers and fractions of various sub-types of NCFs in the Harris dataset.** $k$ is the number of inputs to a BF. The columns under the 'Number of BFs' grouping give the total number of BFs in the Harris dataset ('Total' column) and the numbers of BFs belonging to NCFs, $ChF_0$, $ChF_1$, $ChF_U$ and *non-*$ChF_U$ NCF types for various number of inputs. The columns under the 'Fraction of sub-types within NCFs' grouping give the fraction of the different sub-types of NCFs, namely, $ChF_0$, $ChF_1$, $ChF_U$ and *non-*$ChF_U$ NCF within the NCFs for various number of inputs.

| $k$ | Number of BFs | | | | | | Fraction of sub-types within NCFs | | | |
|---|---|---|---|---|---|---|---|---|---|---|
| | Total | NCF | $ChF_0$ | $ChF_1$ | $ChF_U$ | *non-*$ChF_U$ NCF | $ChF_0$ | $ChF_1$ | $ChF_U$ | *non-*$ChF_U$ NCF |
| 1 | 2 | 2 | 2 | 2 | 2 | 0 | 1.0 | 1.0 | 1.0 | 0.0 |
| 2 | 9 | 9 | 9 | 3 | 9 | 0 | 1.0 | 0.33333 | 1.0 | 0.0 |
| 3 | 71 | 71 | 59 | 3 | 62 | 9 | 0.83099 | 0.04225 | 0.87324 | 0.12676 |
| 4 | 38 | 35 | 26 | 0 | 26 | 9 | 0.74286 | 0.0 | 0.74286 | 0.25714 |
| 5 | 19 | 16 | 11 | 0 | 11 | 5 | 0.6875 | 0.0 | 0.6875 | 0.3125 |

**Table S13.** **Relative enrichment ratios of various sub-types of NCFs within NCFs in the Harris dataset** $k$ is the number of inputs to a BF. $ChF_0$, $ChF_1$, $ChF_U$ and $non\text{-}ChF_U$ NCF correspond to the chain-0, chain-1, generalized chain functions and NCFs that are not generalized chain functions respectively. The columns $ChF_0$, $ChF_1$, $ChF_U$ and $non\text{-}ChF_U$ NCF are the relative enrichment ratios (see Main text, **Methods** section) for the associated sub-types within NCFs in the Harris dataset. $E_R > 1$ indicates that there is an enrichment of the sub-type within NCFs. NA implies that there are no BFs of the type $non\text{-}ChF_U$ NCF in theory.

| $k$ | Relative enrichment ratios ($E_R$) of sub-types within NCFs | | | |
|---|---|---|---|---|
| | $ChF_0$ | $ChF_1$ | $ChF_U$ | $non\text{-}ChF_U$ NCF |
| 1 | 1.0 | 1.0 | 1.0 | NA |
| 2 | 1.33333 | 0.44444 | 1.0 | NA |
| 3 | 2.0455 | 0.10401 | 1.07476 | 0.67606 |
| 4 | 3.64495 | 0.0 | 1.82248 | 0.43408 |
| 5 | 6.75046 | 0.0 | 3.37523 | 0.39243 |

**Table S14.** **Statistical significance of the relative enrichments ($E_R$) of various sub-types of NCFs within NCFs for BFs in the Harris dataset.** $k$ is the number of inputs to a BF. The columns $ChF_0$, $ChF_1$, $ChF_U$ and $non\text{-}ChF_U$ NCF give the $p$-values associated with the relative enrichment see Main text, **Methods** section) of these sub-types of NCFs, within NCFs, for the Harris dataset. $p$-values $< 0.05$ are considered statistically significant. These $p$-values are used to assign the statistical significance stars in Fig. S3. Note that all 2-input NCFs are $ChF_U$s, hence it is meaningless to compute $p$-values for both $ChF_U$ and $non\text{-}ChF_U$ NCF.

| $k$ | $p$-values associated with $E_R$ for sub-types of NCFs | | | |
|---|---|---|---|---|
| | $ChF_0$ | $ChF_1$ | $ChF_U$ | $non\text{-}ChF_U$ NCF |
| 2 | 0 | 0.99001 | NA | NA |
| 3 | $3.17607 \times 10^{-14}$ | 1.00000 | 0.06557 | 0.87972 |
| 4 | $9.14592 \times 10^{-13}$ | 0.99966 | $1.32004 \times 10^{-05}$ | 0.99994 |
| 5 | $1.52758 \times 10^{-09}$ | 0.82068 | $4.04322 \times 10^{-06}$ | 0.99996 |

**Table S15.** **Nodewise enumeration of the number of BFs that satisfy biological constraints for the Pancreas cell differentiation GRN.** ‘Nodes’ are the names of the nodes in the network and $k$ is the associated number of inputs to that node. The columns $ChF_0$, $ChF_1$ and $ChF_U$ give the number of chain-0, chain-1 and generalized chain functions that satisfy biological fixed point constraints and sign conforming constraints at each node of the Pancreas cell differentiation GRN. Imposing $ChF_U$ leads to 3600 Boolean models.

| Nodes | Inputs | $ChF_0$ | $ChF_1$ | $ChF_U$ |
|---|---|---|---|---|
| Pdx1 | 1 | 1 | 1 | 1 |
| Ptf1a | 3 | 4 | 6 | 10 |
| Ngn3 | 3 | 4 | 6 | 10 |
| Pax4 | 3 | 4 | 2 | 6 |
| Arx | 3 | 4 | 2 | 6 |

**Table S16. Nodewise enumeration of the number of BFs that satisfy biological constraints for the RSCN-2020 GRN.** 'Nodes' are the names of the nodes in the network and $k$ is the associated number of inputs to that node. The columns $ChF_0$, $ChF_1$ and $ChF_U$ columns give the number of chain-0, chain-1 and generalized chain functions that satisfy biological fixed point constraints and sign conforming constraints at each node of the RSCN-2020 GRN. Note here that for the ARF10 node, there is no generalized chain function that satisfies the above-mentioned constraints. Thus for ARF10, the allowed BF was restricted to NCF leading to 4 BFs for that node. Imposing $ChF_U$ on all nodes except for ARF10 (for which NCF is imposed) leads to a total of 645120 models.

| Nodes | Inputs | $ChF_0$ | $ChF_1$ | $ChF_U$ |
|---|---|---|---|---|
| CK | 3 | 1 | 0 | 1 |
| ARR1 | 2 | 1 | 1 | 1 |
| SHY2 | 2 | 1 | 1 | 1 |
| AUXIAA | 1 | 1 | 1 | 1 |
| ARF | 1 | 1 | 1 | 1 |
| ARF10 | 3 | 0 | 0 | 0 |
| ARF5 | 6 | 16 | 32 | 48 |
| XAL1 | 1 | 1 | 1 | 1 |
| PLT | 4 | 0 | 1 | 1 |
| AUX | 1 | 1 | 1 | 1 |
| SCR | 3 | 1 | 0 | 1 |
| SHR | 3 | 0 | 1 | 1 |
| MIR166 | 4 | 2 | 0 | 2 |
| PHB | 4 | 7 | 5 | 12 |
| JKD | 3 | 2 | 0 | 2 |
| MGP | 4 | 8 | 2 | 10 |
| WOX5 | 5 | 7 | 0 | 7 |
| CLE40 | 1 | 1 | 1 | 1 |

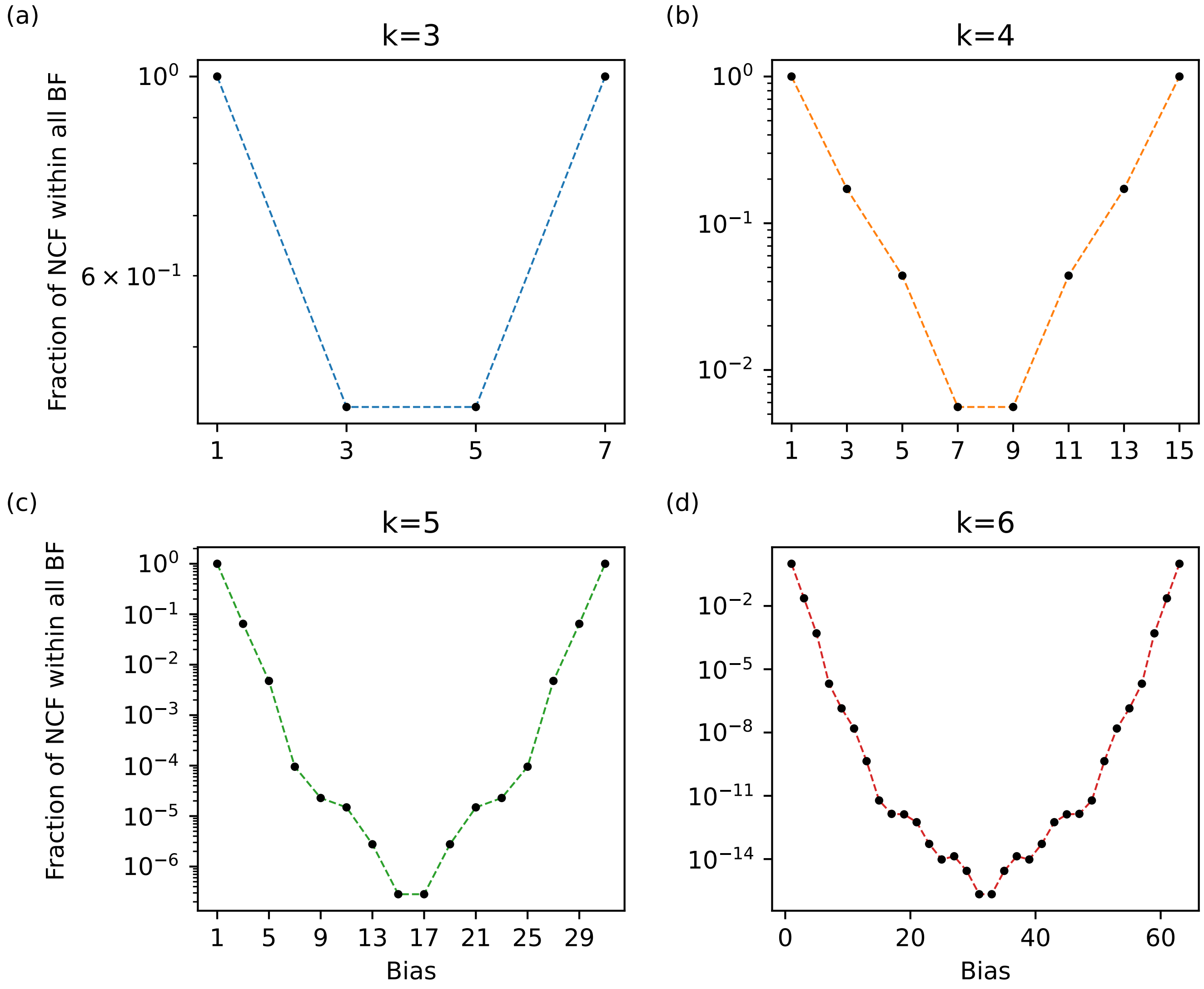


**Figure S1. Bias-wise fraction of NCFs within all BFs.** For a given number of inputs ($k$), the fraction of NCFs in all BFs ($y$-axis) is plotted as a function of the bias ($1 \leq P \leq 2^k - 1$ for odd $P$) ($x$-axis). Subplots correspond to different number of inputs $k = 3, 4, 5$ and 6.

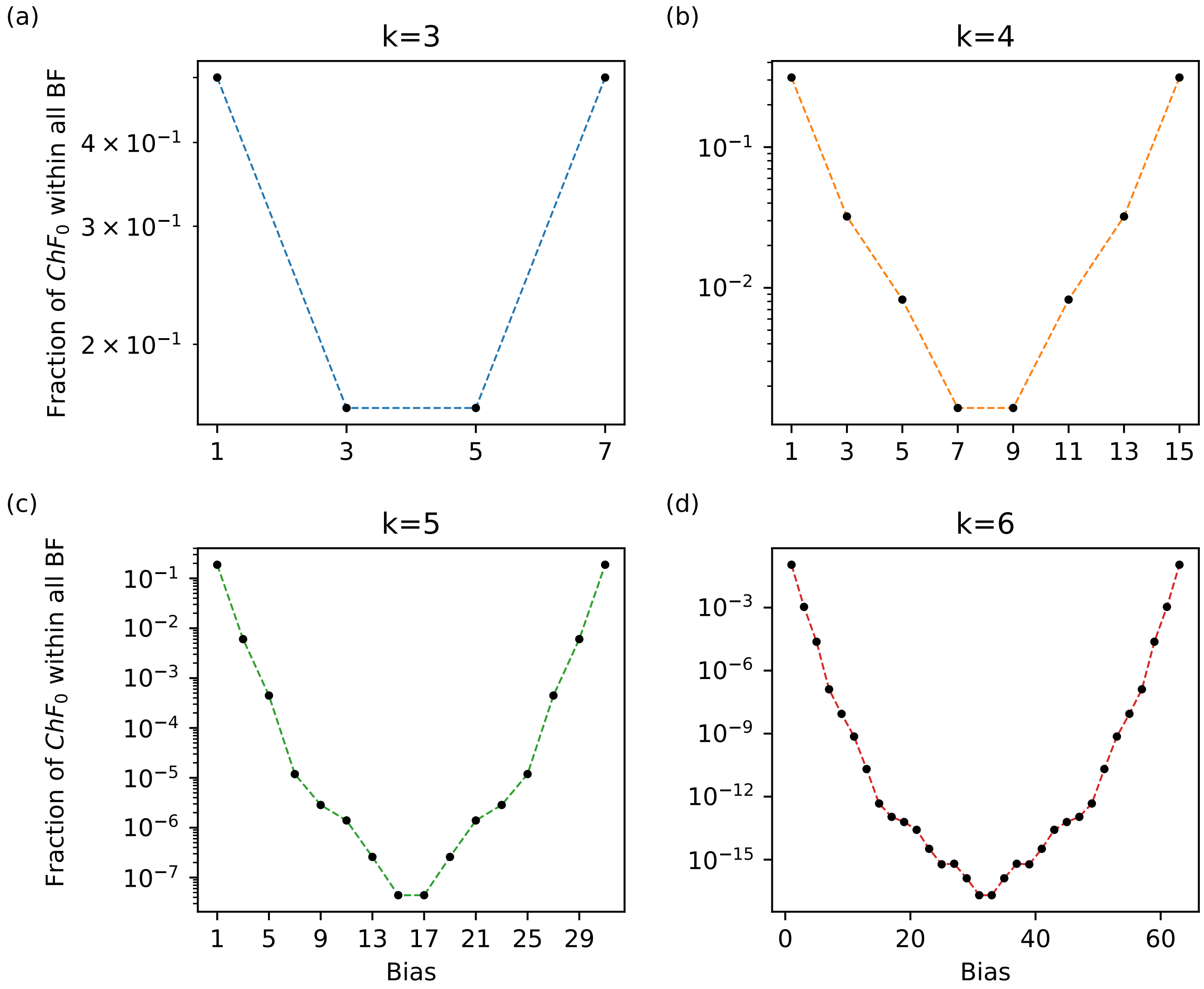


**Figure S2.** **Bias-wise fraction of chain-0 (or chain-1) within all BFs.** For a given number of inputs ($k$), the fraction of $ChF_0$ (or $ChF_1$) in all BFs ($y$-axis) is plotted as a function of the bias ($1 \leq P \leq 2^k - 1$ for odd $P$) ($x$-axis). Subplots correspond to different number of inputs $k = 3, 4, 5$ and 6.

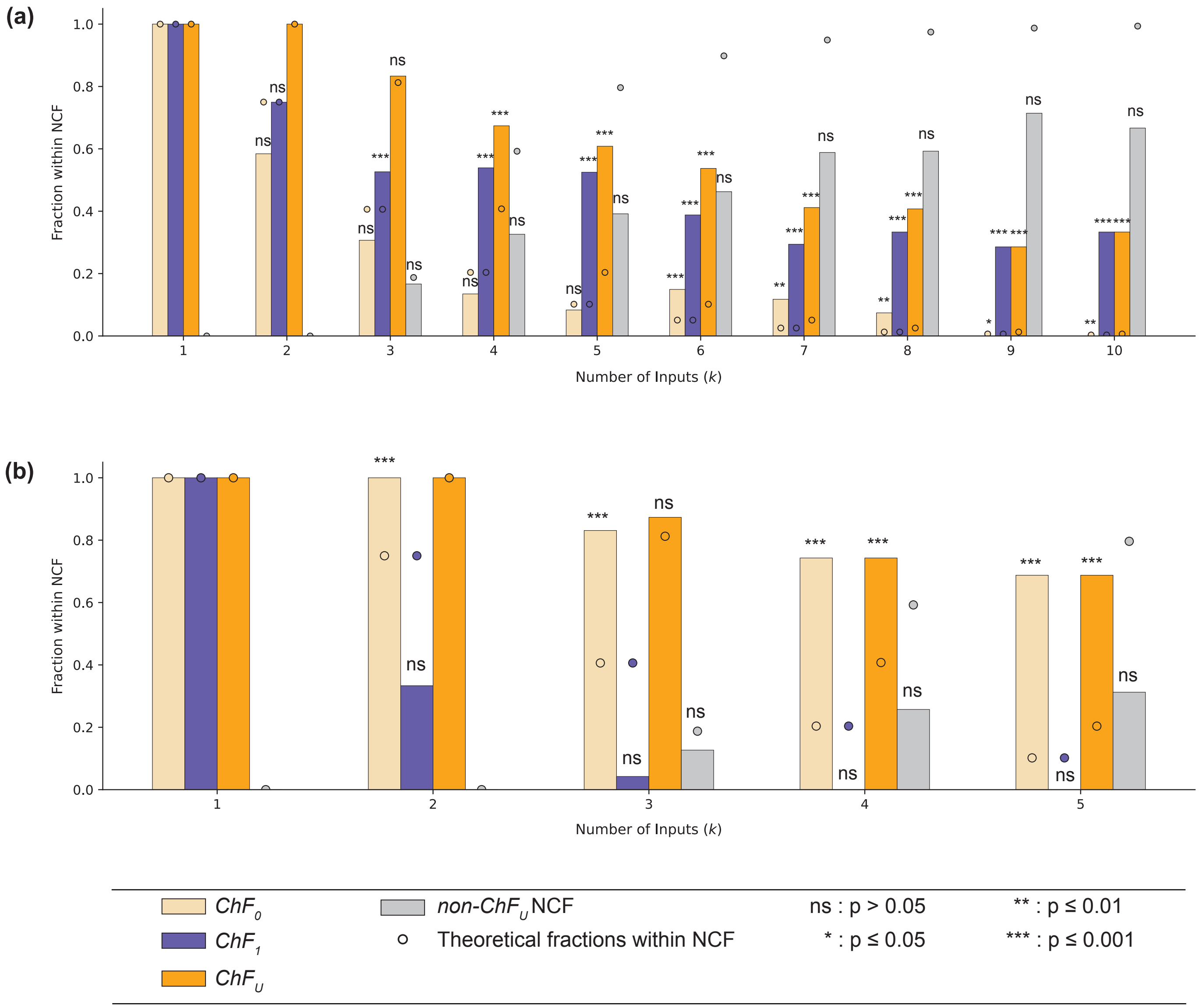


**Figure S3. Fractions of various sub-types of NCF within NCFs in the MCBF dataset and Harris dataset (a)** This sub-figure shows the fraction of $ChF_0$, $ChF_1$, $ChF_U$ and *non-*$ChF_U$ NCF within NCFs, in theory and in the MCBF dataset as dots and colored bars respectively. The relative enrichments of $ChF_1$s and $ChF_U$s within NCFs are statistically significant for $k \geq 3$ and $k \geq 4$ respectively. **(b)** This sub-figure shows the fractions of $ChF_0$, $ChF_1$, $ChF_U$ and *non-*$ChF_U$ NCF within NCFs in theory and in the Harris dataset as dots and colored bars respectively. The relative enrichments of $ChF_0$s and $ChF_U$s within NCFs are statistically significant for $k \geq 2$ and $k \geq 4$ respectively.
